# Magnetic resonance reveals early lipid deposition in murine prediabetes as predictive marker for cardiovascular injury

**DOI:** 10.1038/s44303-024-00044-0

**Published:** 2024-09-23

**Authors:** Katja Heller, Vera Flocke, Tamara Straub, Zhaoping Ding, Tanu Srivastava, Melissa Nowak, Florian Funk, Bodo Levkau, Joachim Schmitt, Maria Grandoch, Ulrich Flögel

**Affiliations:** 1 Institute for Translational Pharmacology, Medical Faculty, University Clinics and Heinrich Heine University, Düsseldorf, Germany; 2 Experimental Cardiovascular Imaging, Institute for Molecular Cardiology, Heinrich Heine University, Düsseldorf, Germany; 3 Institute for Pharmacology, Heinrich Heine University, Düsseldorf, Germany; 4 Institute for Molecular Medicine III, Heinrich Heine University, Düsseldorf, Germany; 5 Cardiovascular Research Institute Düsseldorf (CARID), Heinrich Heine University, Düsseldorf, Germany

**Keywords:** Imaging techniques, Molecular imaging, Magnetic resonance imaging, NMR spectroscopy

## Abstract

People with diabetes have an increased cardiovascular risk and a poorer outcome after myocardial infarction (MI). However, the exact underlying mechanisms are still unclear, as is the question of which non-invasive measures could be used to predict the altered risk for the patient at early stages of the disease and adapt personalized treatment. Here, we used a holistic magnetic resonance approach to monitor longitudinally not only the main target heart, but also liver, peripheral/skeletal muscle, bone marrow, and hematopoiesis during disease development and subsequent MI. In prediabetic mice, we found a strong accumulation of lipids in all organs which preceded even a significant whole-body weight gain. Intramyocellular lipids (IMCLs) were most sensitive to reveal in vivo very early alterations in tissue properties during the prediabetic state. Subsequent induction of MI led to a persistent impairment of contractile function in septal/posterior segments of prediabetic hearts which correlated with their lipid load prior MI. At the same time, prediabetic cardiomyocytes exhibited sarcomere function at its limit resulting in overload and lower compensatory contractility of the healthy myocardium after MI. In summary, we identified IMCLs as very early marker in murine prediabetes and together with the cardiac lipid load as predictive for the functional outcome after MI.

## Introduction

Diabetes mellitus is a major risk factor for the development of cardiovascular disease (CVD) making CVD a leading comorbidity in patients with type 2 diabetes (T2D)^1^. Indeed, T2D is known to increase the risk of heart failure^2^ and to worsen outcomes after acute myocardial infarction (MI)^3^. Although the effects of hyperglycemia and diabetes on myocardial ischemia-reperfusion (I/R) injury have been extensively studied, the underlying mechanisms remain elusive—and with this also potential non-invasive readouts (blood parameters, imaging-derived measures) which could be used to predict the altered risk for the patient and to adapt personalized treatment. While human data suggest that diabetic hearts are more susceptible to acute I/R injury^4^, experimental animal studies have been less consistent^5^ whereby also the depth of phenotyping critically determines the validity of the obtained results. Thus, it is of crucial importance to carry out a comprehensive characterization of the used model with special focus on very early manifestations of the disease, when timely therapeutic interventions might completely reverse the initial pathological alterations.

An established model for studying the early stages of obesity-related pathologies is the exposure of male C57BL/6J mice to a long-term high-sugar/high-fat diabetogenic diet (DD)^6,7^. Over time, this results in mice with diet-induced obesity exhibiting common prediabetic characteristics such as hyperglycemia, impaired insulin sensitivity, and low-grade chronic inflammation^8^. Pathophysiologically, this prediabetic state closely resembles the human situation, also characterized by developing insulin resistance, increased glucose levels, and the onset of inflammation^9,10^. Since, the dysregulated insulin response of heart, liver, and muscle, is usually accompanied by a systemic immune response^11^, we hypothesized that this might detrimentally impact on the healing process after MI.

To investigate early processes of diabetes development and progression as well as possible effects on the outcome after cardiac I/R, in the present study we used a combination of both diseases in that we applied a widely used mouse model of myocardial I/R injury to prediabetic mice^6^^,10^. In order to identify incipient alterations and potential biomarkers for decision-making, we employed a holistic approach by longitudinally monitoring important organs/tissues by non-invasive magnetic resonance imaging and spectroscopy (MRI/MRS). With this, we mapped the entire process from health and development of the prediabetic state, acutely induced cardiac injury, and subsequent healing with regard to disturbed metabolism, immune response, and cardiac function. This encompassed in particular the main target heart, but also liver, peripheral/skeletal muscle, and bone marrow from baseline to 9 weeks of DD, induction of MI, and 3 weeks follow-up. These analyses were completed by invasive measures of bone marrow fat, hematopoiesis, circulating immune cells, but also calcium handling using histology, flow cytometry, and sarcomere measurements at selected time points.

## Results

For induction of prediabetes, we used an established model with exposure of male 12-week-old C57BL/6J mice to a high-caloric, high-fat diabetogenic diet (DD) for a period of 9 weeks^6^. Age- and weight-matched mice C57BL/6J mice which were fed a regular chow diet served as control. After 9 weeks of diet, mice were subjected to cardiac I/R injury, while diet was maintained until 21 days after IR. Intraperitoneal glucose tolerance tests (i.p. GTT) and fasting blood glucose levels corroborated development of a prediabetic phenotype after 9 weeks of feeding (Supplementary Fig. 1a, b). Upon I/R, fasting blood glucose levels remained elevated and glucose tolerance impaired (Supplementary Fig. 1c, d) indicating maintenance of sustained metabolic stress during cardiac I/R and the subsequent healing phase. Consistently, body weight, insulin tolerance, and plasma insulin levels were adversely affected in DD-fed mice (Supplementary Fig. 1e–g).

To assess the functional, metabolic, and immunological impact of diet and MI, mice were subjected at baseline, 3, 6, and 9 weeks of feeding as well as 1, 3, 7, and 21 days post MI to comprehensive, non-invasive MRI/MRS analyses completed by histology, flow cytometry, and sarcomere measurements at selected time points. Please note, due to animal welfare and to avoid prolonged or several periods of anesthesia, individual mice were not subjected to all MRI/MRS measurements at respective time points. Data were compiled from different experimental series where mice usually received either thoracic (cardiac and liver) or hindlimb (tibialis muscle and bone marrow) measurements at individual time points. We first summarize the DD-specific alterations compared with the control diet and then the associated consequences after MI.

### Organ-specific lipid load by DD

**Fig. 1:**
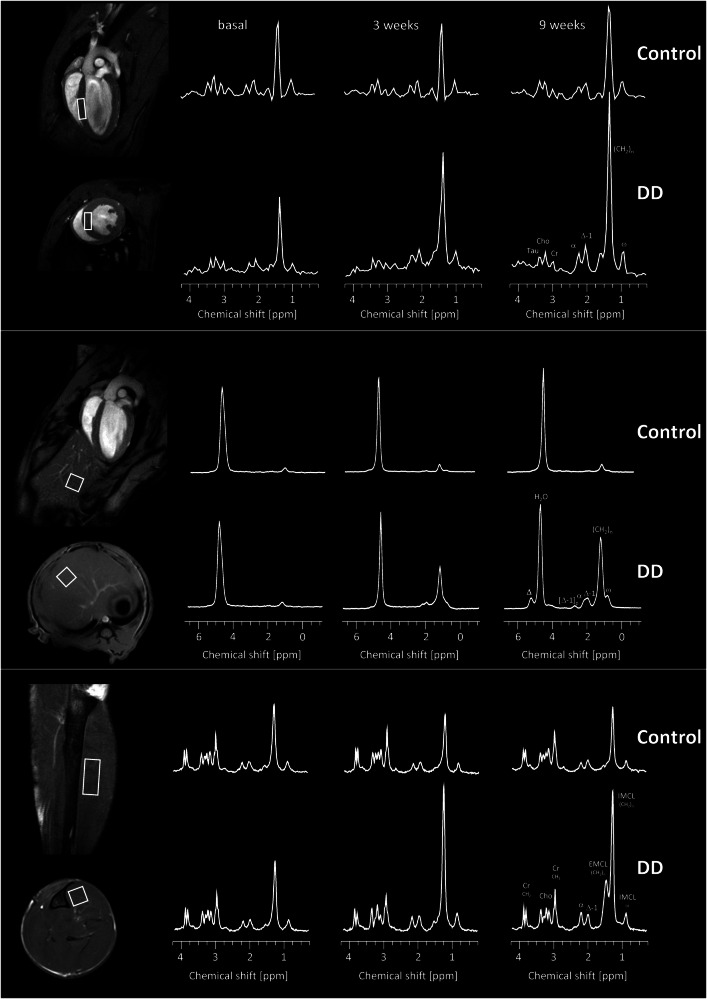
Volume-selective ^1^H MRS to assess organ-specific lipid contents over time. Characteristic ^1^H MR spectra from heart (top), liver (middle), and tibialis anterior muscle (bottom) under control diet (upper rows) and DD (lower rows) at baseline (2nd column), 3 weeks (3rd column) and 9 weeks (4th column) of feeding. The white rectangles within the anatomic ^1^H MR reference images indicate the localization of the spectroscopic voxels for the individual organs (1st column). Abbreviations: Cho, choline; Cr, creatine; Tau, taurine; α, Δ, Δ-1, [Δ-1], ω and (CH_2_)_n_ indicate the respective positions within the carbon chain of the triglycerides.

To explore the metabolic impact of the diet on a tissue-specific level over time, we used volume-selective ^1^H MRS to assess the lipid content in organs known to be critically affected by diabetes, i.e., heart, liver, and skeletal muscle. To this end, small voxels (white rectangles, Fig. 1, 1st column) were carefully placed in each organ, taking care not to overlap with surrounding tissue. For cardiac lipids, the spectroscopic voxel was localized in the interventricular septum (Fig. 1, top) to prevent signal contaminations from peri/epicardial fat. Furthermore, it is the site where the least movement of the myocardium occurs over the entire cardiac cycle, thus reducing potential motion artefacts (see Supplementary Fig. 2 for a detailed explanation of voxel localization and timing of data acquisition). Hepatic lipids were determined from a voxel in the left liver lobe with proper distance to large blood vessels (Fig. 1, middle) and skeletal muscle lipids from the *Musculus tibialis anterior* (Fig. 1, bottom). The latter offers the unique opportunity that extra- and intramyocellular lipids (EMCLs, IMCLs) can be spectrally separated when the muscle fibers are aligned along the main magnetic field direction^12^.

**Fig. 2:**
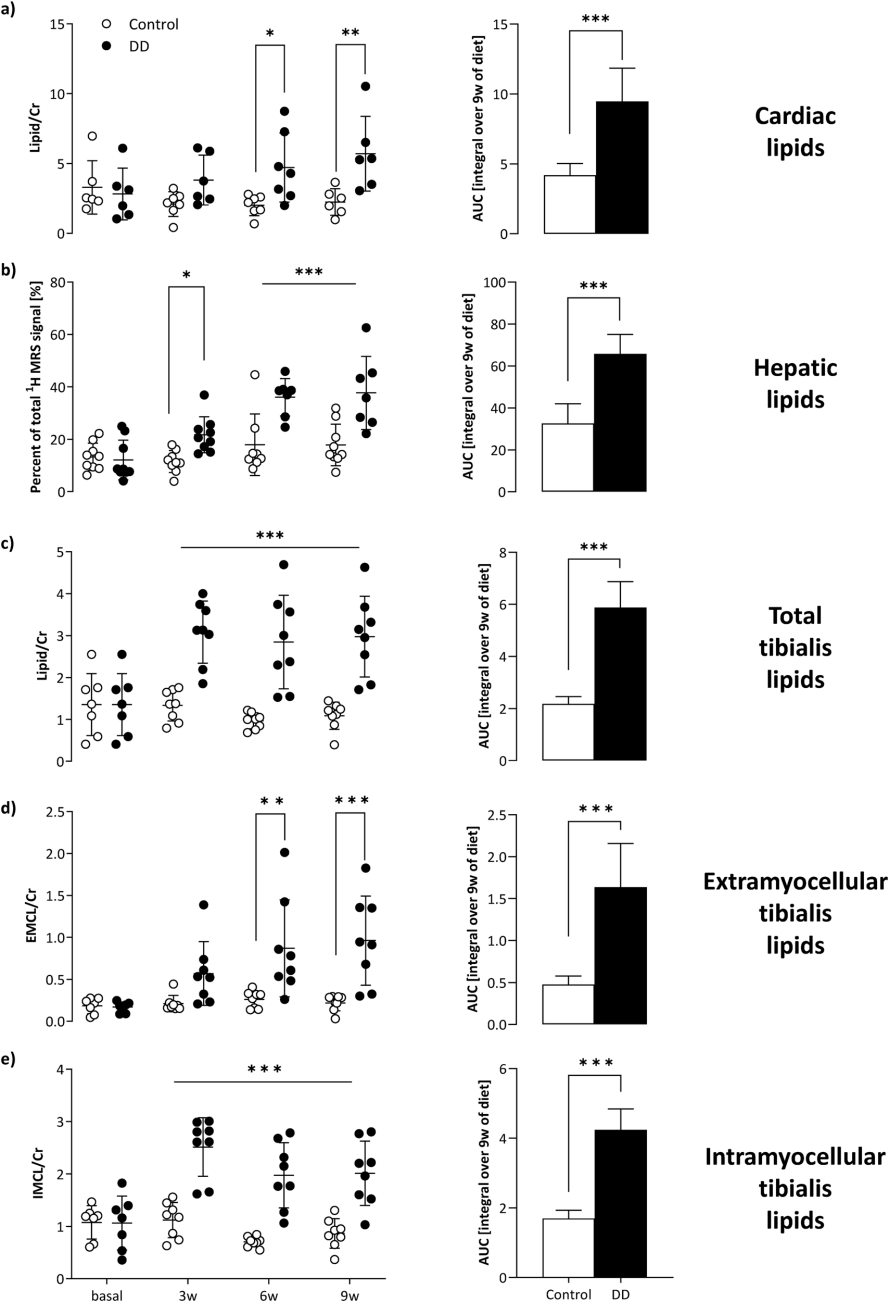
Tibial IMCL as early and sensitive marker of prediabetes. Quantification of in vivo ^1^H MRS lipid levels with individual data points (left) and area under the curve (AUC, right), i.e., the integral over 9 weeks for control diet (white) and DD (black) for heart (**a**), liver (**b**), and skeletal muscle (tibialis anterior; total (**c**), EMCL (**d**), IMCL (**e**). Cardiac and skeletal muscle lipid levels were related to the respective creatine signals, while hepatic lipids are given as percent of the total ^1^H signal (including water); *n* = 6–8, *P ≤ 0.05, **P ≤ 0.01, ***P ≤ 0.001.

Representative baseline ^1^H MR spectra are illustrated in Fig. 1, 2nd column, for each organ. Of note, in spectra from heart and tibialis muscle, the dominating water signal was suppressed to allow accurate identification of the low lipid signals. With this, resonances for creatine, taurine, choline, and several signals originating from lipids (see Figure legend for more details) could be unequivocally resolved (Fig. 1, top+bottom). While signals for the first metabolites were unaltered during the course of the diet, all lipid peaks were clearly increased in DD mice as compared to control animals (Fig. 1, 3rd and 4th column). Quantification of the in vivo data (Fig. 2) revealed that the susceptibility of the investigated organs to the diabetogenic diet was largely differential: interestingly, the strongest response upon onset of DD feeding was observed for lipids in the tibialis muscle (Fig. 2c). Here, IMCLs exhibited already a massive increase after 3 weeks of DD (Fig. 2e), staying almost constant thereafter, while EMCLs (Fig. 2d) increased steadily over time. Of note, the approximately three-fold increase in tibialis IMCLs preceded even a significant weight gain in DD-fed mice (Supplementary Fig. 1e) and proved to be the most sensitive parameter for the induced metabolic changes. These very early alterations in skeletal muscle were followed by a substantial hepatosteatosis after 6 weeks of feeding (Fig. 2b) and also a significant elevation of cardiac lipids in DD animals (Fig. 2a). Despite the almost three-fold fat increase in the heart, functional cardiac parameters were unaffected over the 9 weeks of diet (Supplementary Fig. 3).

### DD impacts on bone marrow fat and hematopoiesis

**Fig. 3:**
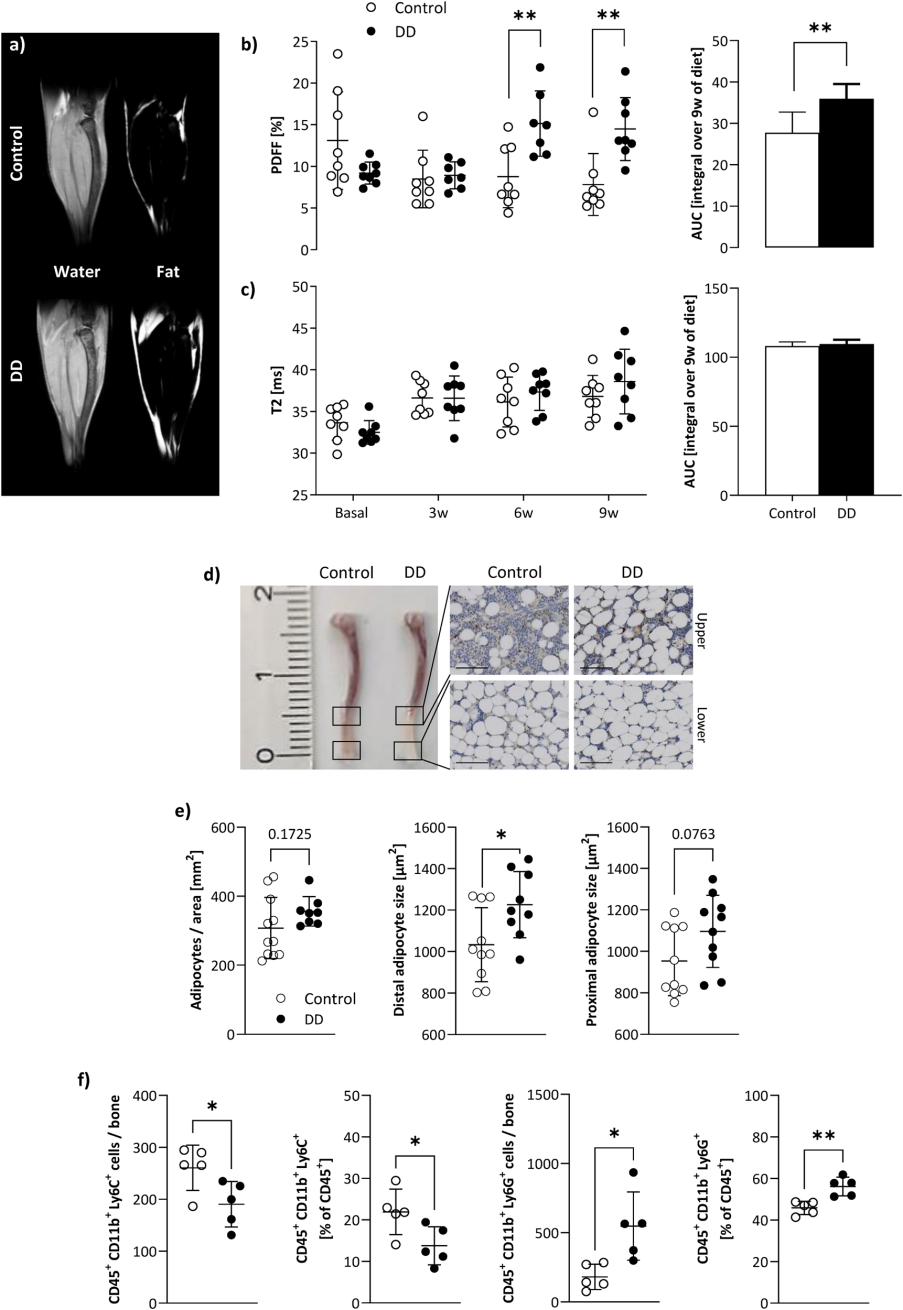
Impact of DD on tibial bone marrow fat and hematopoiesis. **a** ^1^H MRI water/fat separation by multi-chemical shift selective imaging. Quantification of tibial bone marrow PDFF (**b**) and T2 (**c**) over time (left) as well as the AUC (right; i.e., the integral over 9 weeks of diet); white = control, black = DD; *n* = 8, each group. **d** Post-mortem confirmation of the in vivo data after 9 weeks of DD versus control diet by representative images of murine tibiae and histological slices of upper and lower distal parts of H&E-stained bones (40× magnification; scale bar = 100 µm). **e** Quantification of histology with number of bone marrow adipocytes per normalized area (left) and adipocyte size in the distal (middle) and proximal (right) part of the tibia of DD-fed mice versus chow-fed mice (*n* = 8–11). **f** Flow cytometric analysis of bone marrow monocytes (CD45^+^ CD11b^+^ Ly6C^+^; 1st + 2nd column) and neutrophils (CD45^+^ CD11b^+^ Ly6G^+^; 3rd + 4th column) in absolute numbers per bone and normalized to CD45^+^ leukocytes [%], respectively; *n* = 5 each, *P ≤ 0.05, **P ≤ 0.01.

Given the substantial alterations in organ lipid content described above, we further focused on the bone marrow as a hematopoietic niche crucially involved in the immune response prior and post-MI. Since spectroscopic approaches for the murine bone marrow are somewhat hampered due to the small voxel sizes required, we used here water/fat separation by multi-chemical shift selective imaging (mCSSI^13^) to determine the bone marrow proton density fat fraction (PDFF) and employed T2 mapping for additional tissue characterization. As can be recognized from representative water/fat images from the calf (Fig. 3a), diabetogenic feeding resulted in a substantial fat accumulation particularly in the distal part of the tibia, which reached the level of significance after 6 weeks of DD (Fig. 3b). In contrast to the pronounced lipid increase in the bone marrow of DD mice detected by PDFF measurements, parametric maps (T2) of the tibia provided no evidence for any alterations in bone marrow tissue texture (Fig. 3c). Of note, the same was true for the adjacent tibialis muscle (Supplementary Fig. 4), so the direct chemical-shift-based approaches (either mCSSI for the bone marrow and ^1^H MRS for the muscle) proved most sensitive to these rapid metabolic effects on DD exposure here.

Consistent with the DD-related fat accumulation observed in vivo, histology revealed an increased number as well as size of adipocytes in the tibia, especially in the distal part, due to DD feeding (Fig. 3d, e). In separate experiments, flow cytometry was used to verify whether the raised fat fraction of the bone marrow was also associated with alterations in immune cell content. Of note, we found opposing results for monocytes and neutrophils isolated from the tibia: while total number and proportion of monocytes were decreased after 9 weeks of DD as compared to control mice (Fig. 3f, left), both measures were increased for neutrophils (Fig. 3f, right). Interestingly, this effect was not exactly reflected in the blood. Parallel analysis of circulating immune cells revealed raised values for both cell populations (Supplementary Fig. 5), suggesting a generally enhanced systemic immune response in DD compared to control mice.

### Distinct lipolysis patterns after I/R

**Fig. 4:**
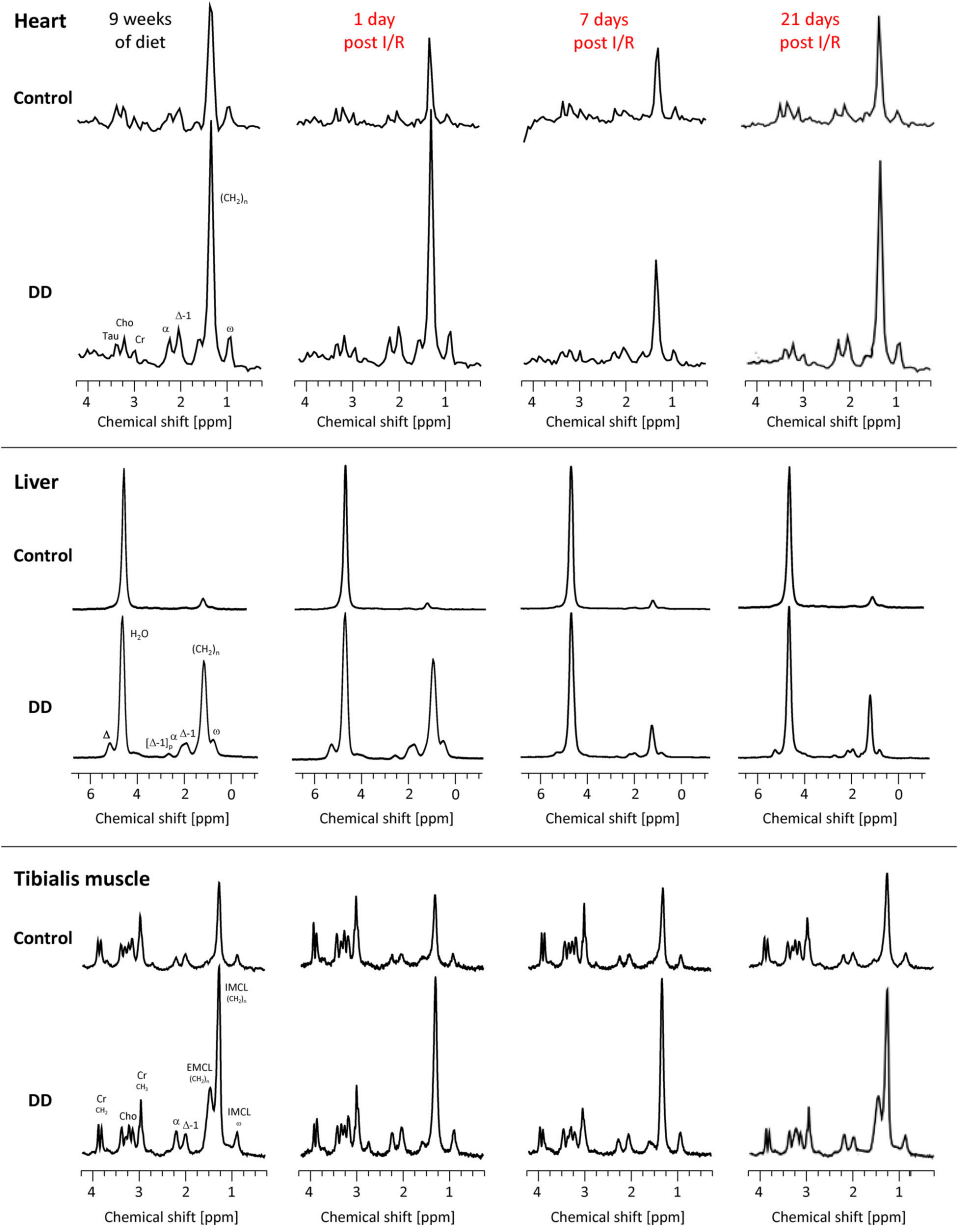
Volume-selective ^1^H MRS to monitor organ-specific lipid contents after I/R. Characteristic ^1^H MR spectra from heart (top), liver (middle), and tibialis anterior muscle (bottom) under control diet (upper rows) and DD (lower rows) after 9 weeks of feeding just prior induction of I/R (1st column), 1 day after I/R (2nd column) as well as 7 and 21 days after I/R (3rd and 4th column, respectively). For localization of the spectroscopic voxels please refer to Fig. 1. Abbreviations: Cho choline; Cr creatine; Tau taurine; α, Δ, Δ-1, [Δ-1], ω and (CH_2_)_n_ indicate the respective positions within the carbon chain of the triglycerides.

The massive sympathetic stimulation upon cardiac I/R is expected to trigger systemic lipolysis. Interestingly, volume-selective ^1^H MRS during the course after MI (Fig. 4) revealed that—at least in case of DD—lipid pools in the organs investigated were affected differentially by this stimulus: peripheral EMCL depots were most sensitive to this, in that they dropped already at day 1 after I/R to baseline levels before initiation of DD (Fig. 4 bottom and 5d), which was followed by an almost complete depletion of the overloaded lipid pools in heart and liver until day 7 post-MI (Fig. 4 top + middle and 5a, b). However, with progressive recovery from the insult, organ lipid levels tended again to rise in mice further maintained at DD (Figs. 4 + 5). Of note, only IMCLs in skeletal muscle and the bone marrow PDFF appeared to be unaffected by the systemic response and remained as elevated as prior induction of I/R (Figs. 4 bottom and 5e, f).

**Fig. 5:**
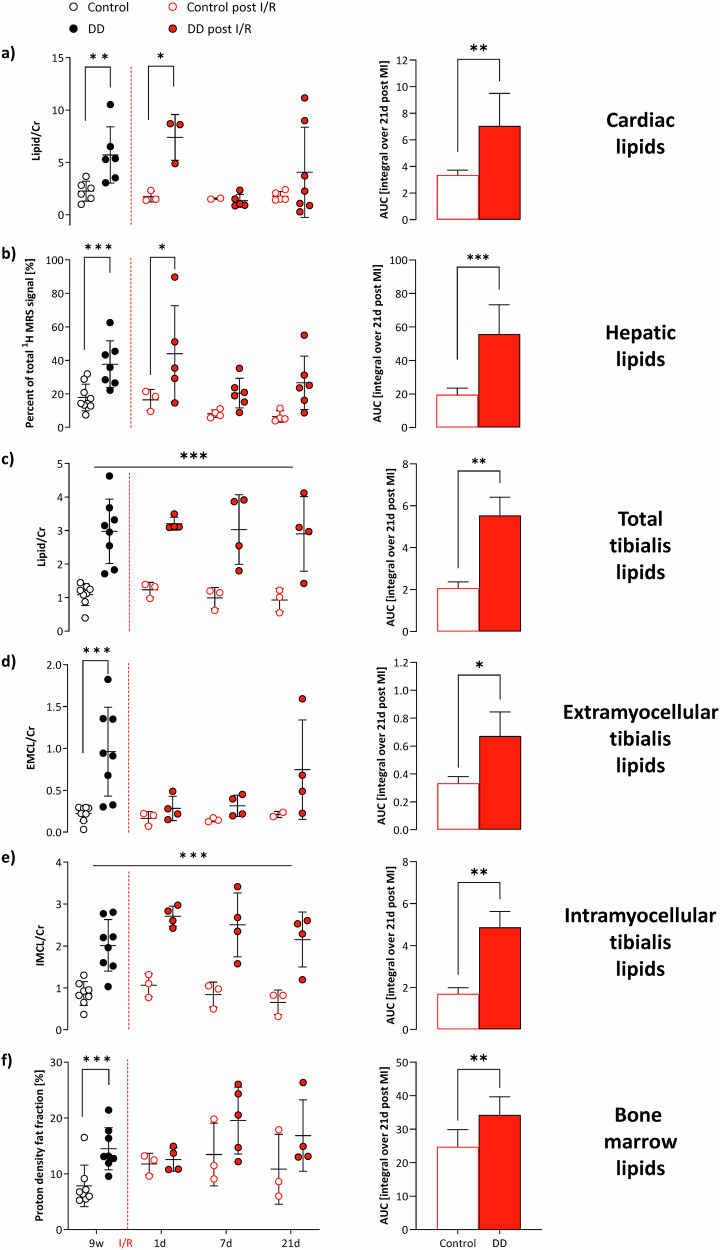
Differential organ lipolysis patterns after I/R. Left, Quantification of lipids from localized in vivo ^1^H MR spectra after I/R (red) from day 1 until 3 weeks after I/R compared to levels prior to the insult after 9 weeks of feeding (black) with open symbols for control diet and closed symbols for DD and right, AUC (i.e., the integral over 21 days post-MI) for heart (**a**), liver (**b**), skeletal muscle (tibialis anterior; total (**c**), EMCL (**d**), IMCL (**e**)) and bone marrow (**f**). Cardiac and skeletal muscle lipid levels were related to the respective creatine signals, while hepatic and bone marrow lipids are given as percent of the total ^1^H signal; *n* = 3–8, *P ≤ 0.05, **P ≤ 0.01, ***P ≤ 0.001.

### Minor impact of DD on global cardiac function and immune response after I/R

**Fig. 6:**
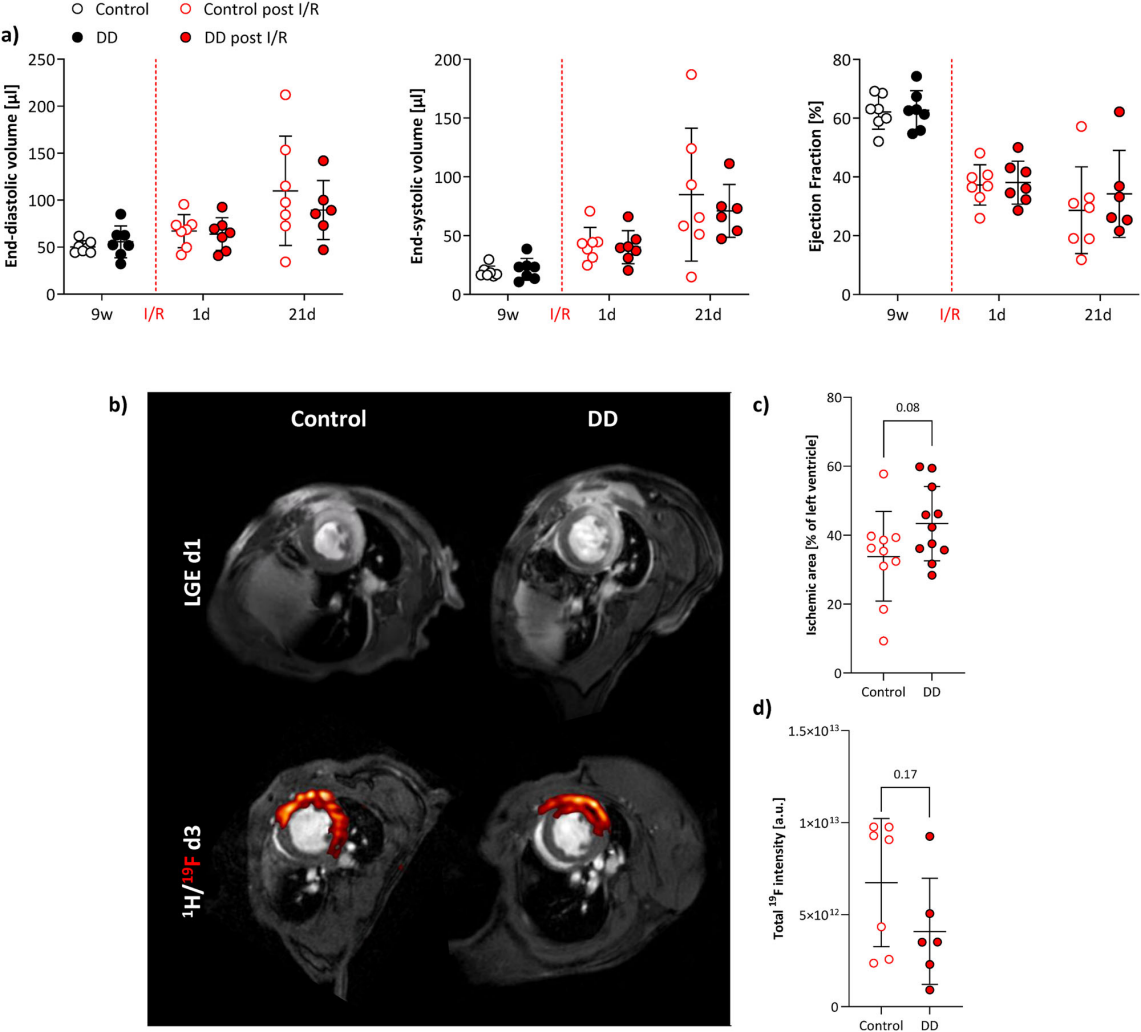
Minor impact of DD on global function and immune response after I/R. **a** Quantification of global EDV (left), ESV (middle), and EF (right) from cine ^1^H MR movies after I/R (red) compared to values prior to the insult after 9 weeks of feeding (black) with open symbols for control diet and closed symbols for DD (*n* = 6–7). **b** Representative examples of LGE enhancement at day 1 (top) and subsequent ^1^H/^19^F MRI at day 3 after I/R (bottom) showing congruent patterns of LGE-positive and inflamed myocardium in control (left) and DD (right) mice. **c** Quantification of LGE-positive myocardium at day 1 after I/R in DD compared to control mice (*n* = 10–11). **d** Integral of ^19^F MR signals in control and DD mice at day 3 after I/R (*n* = 6–7); *P ≤ 0.05.

Functional analysis from cine ^1^H MRI, at first glance, did not indicate any adverse impact of DD on the outcome after MI: both groups showed a similar impairment in functional parameters immediately after the insult and also at the end of the observation period (Fig. 6a). On the other hand, we initially observed a trend towards larger myocardial areas affected by late gadolinium enhancement (LGE) on day 1 after I/R in DD mice (Fig. 6b, c), but which did not reach the level of significance. In order to monitor a putative associated promotion of cardiac inflammation, we employed ^1^H/^19^F MRI in combination with perfluorocarbon nanoemulsions (PFCs) for in vivo inflammation imaging^14,15^. To this end, PFCs were injected intravenously 1 day after I/R and 2 days prior the MRI session to allow for adequate ^19^F-loading of circulating immune cells and their subsequent infiltration into the injured myocardium^16^. ^1^H/^19^F MRI was carried out at day 3 after MI with the suspected peak of monocyte recruitment. The detected ^19^F signals matched closely the previously observed LGE patterns (Fig. 6b), but surprisingly we detected only a weak not significant trend to lower ^19^F signals in DD-fed animals as compared to controls (Fig. 6d). In line with this, we found unaltered levels of circulating neutrophils and monocytes at day 1 and 3 post I/R in the blood of mice under DD (data not shown). Obviously, it seems that DD causes disturbed hematopoiesis under ‘plain’ feeding conditions (Fig. 3, Supplementary Fig. 5), but this however does not appear to affect immune cell release into the blood and subsequent recruitment into the infarcted myocardium after I/R.

### Impaired remote function in DD mice post I/R correlates with lipid load prior I/R

**Fig. 7:**
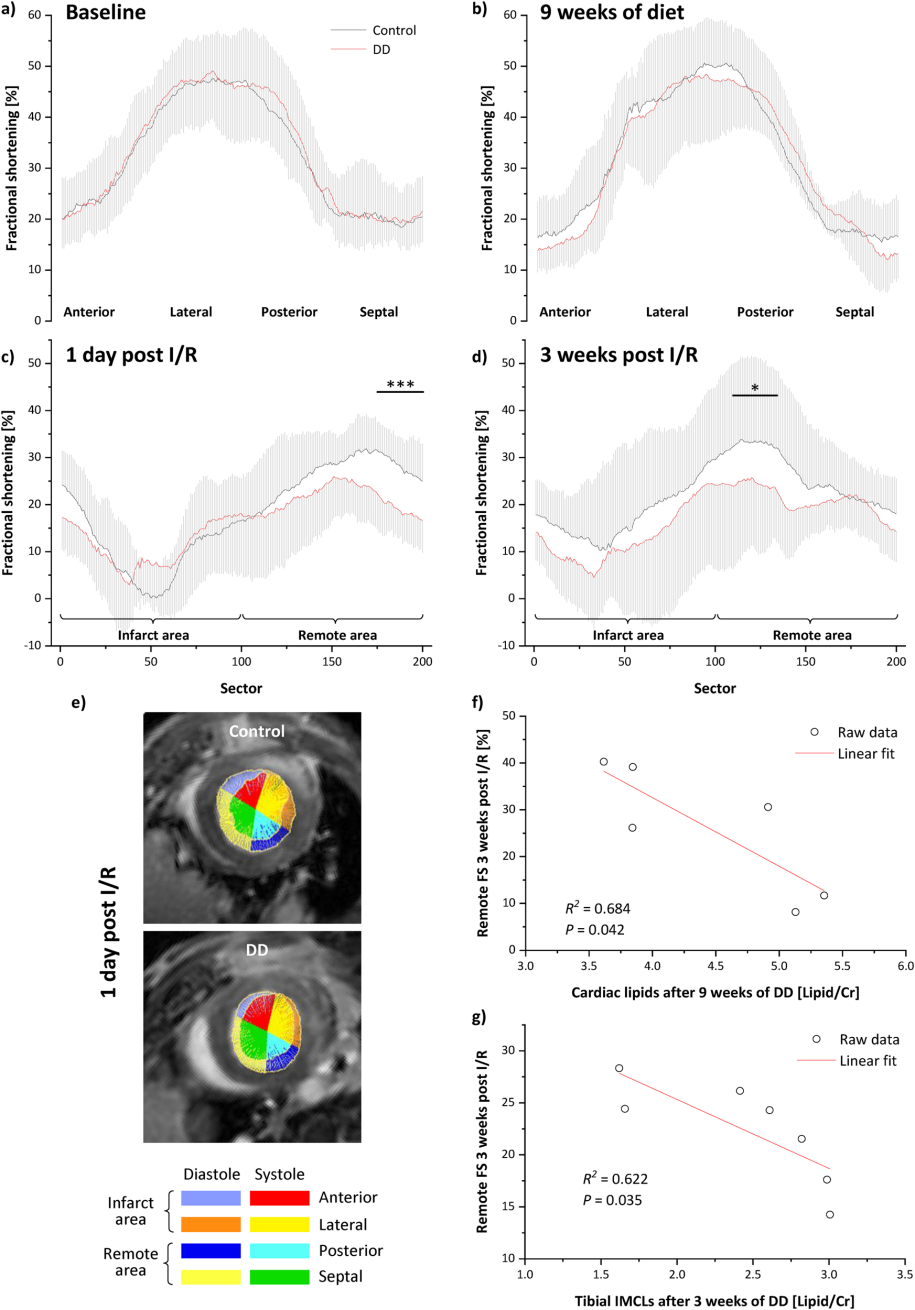
Dysfunctional remote myocardium after I/R depends on lipid load prior I/R. Sectoral analysis of local left ventricular function during the diet and after I/R (see Supplementary Fig. 6 for a detailed description of the applied procedures; for the sake of clarity, SDs are shown within the full sector plots as shadows; *n* = 7–10, *P ≤ 0.05). Similar to global function, DD also did not lead to any regional impairment after 9 weeks of feeding (**a**, **b**). After induction of I/R, massively depressed local fractional shortening (FS) was observed in the ischemic sectors of the anterior and lateral left ventricles in both groups. but, importantly, only in control mice (black) this was accompanied by a compensatory increase in FS in the postero-septal sectors (**c**, **d**). In DD mice (red), FS in the remote areas was significantly decreased throughout the entire observation period post I/R, as assessable from the distance between the diastolic and systolic endocardial radii in the representative images (**e**). **f** Linear regression of remote FS 3 weeks post I/R and cardiac lipid levels immediately prior I/R revealed a significant negative correlation in DD mice. **g** Linear regression of remote FS 3 weeks post I/R and tibial IMCLs after 3 weeks of DD also showed a significant negative correlation in DD mice. Please note, correlations in (**f**, **g**) were derived from separate experimental series.

While global parameters did not provide evidence of increased vulnerability of DD mice to MI (Fig. 6a), a comprehensive sectorial analysis revealed distinct differences in regional myocardial function (cf. Supplementary Fig. 6 for a detailed description of the applied procedures). Specifically, 1 day after I/R, DD-fed mice exhibited impaired compensatory contractility in the remote area with septal fractional shortening (FS) significantly reduced compared to control mice (Fig. 7c), as evidenced by the distance between the diastolic and systolic endocardial radii in the representative images (Fig. 7e). Importantly, although there was no indication of such constraint under baseline conditions and after 9 weeks of feeding (Fig. 7a, b), the impaired remote function persisted until the end of the observation period 3 weeks after I/R (Fig. 7d). Of note, linear regression of cardiac lipid levels immediately prior I/R and the remote FS 3 weeks post I/R revealed a significant negative correlation in the DD group (Fig. 7f). Interestingly, a similar finding was obtained when correlating tibial IMCL levels after 3 weeks of DD—at this early time, the most sensitive metabolic prediabetes marker—with the final remote function 3 weeks after I/R (Fig. 7g).

In order to verify whether the lipid pool detectable by ^1^H MRS (mainly triglycerides) can serve as a surrogate marker for other lipid species that are lipotoxic, in independent experiments we conducted lipidomic profiling of cardiac tissue samples at the end of the initial feeding period just prior induction of MI. Here, we found that hearts from DD versus normal control mice exhibited a distinct accumulation of several ceramide species implicated in the pathogenesis of both T2DM and heart failure^17^. In particular, ceramides C14:0, C18:0, C18:1, and C24:1 were increased, whereas ceramides C16:0, C20:0, C22:0, and C24:0 were unchanged (Supplementary Fig. 7). Of note, ceramide C24:1 is one of the ceramides that most consistently have been associated with heart failure in many different studies^17^.

### Depressed compensatory sarcomere function of prediabetic cardiomyocytes upon MI

**Fig. 8:**
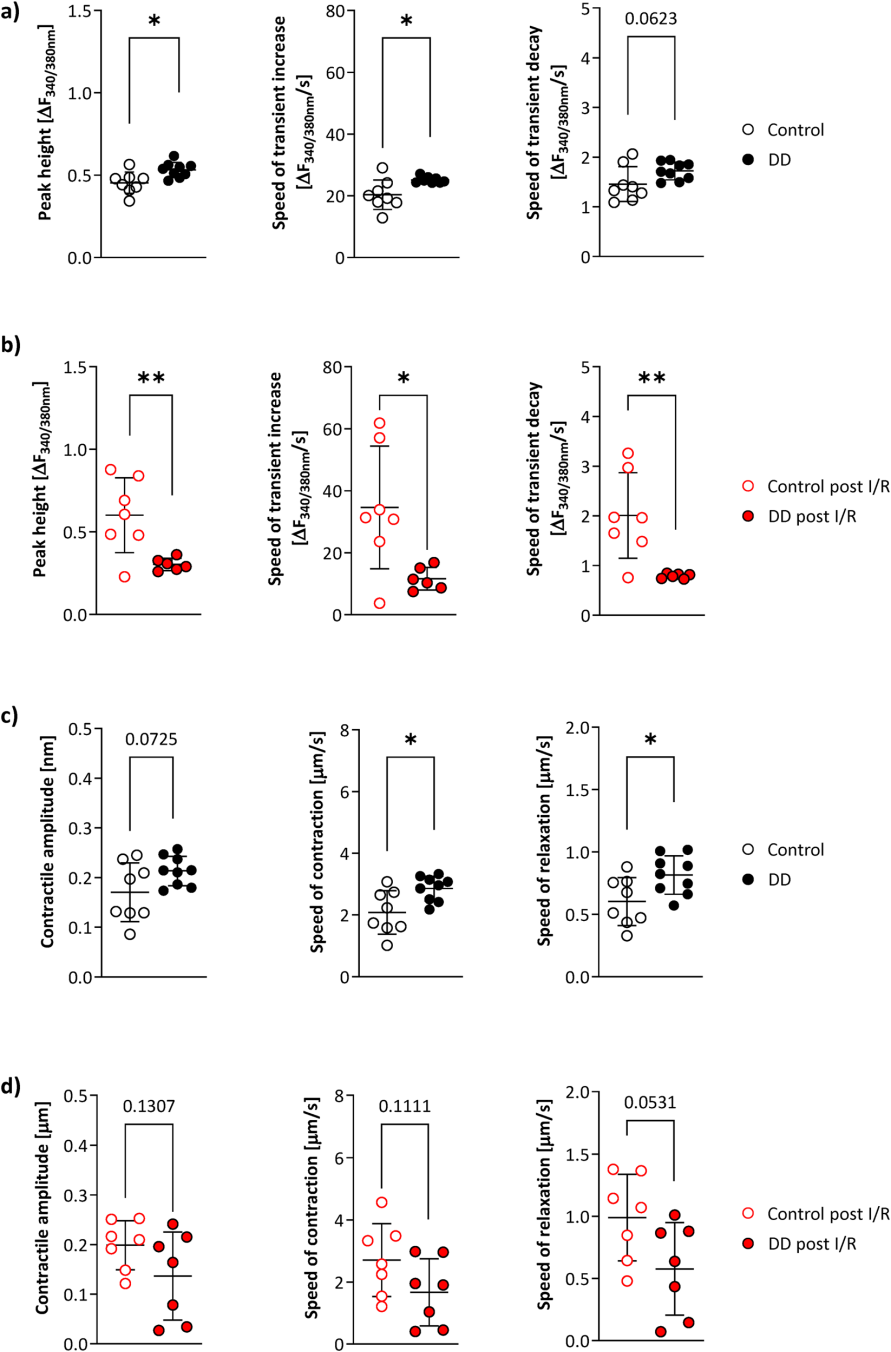
Impaired remote calcium cycling and sarcomere function in DD mice after I/R. Calcium cycling in isolated cardiomyocytes of DD (closed symbols) and control (open symbols) mice after (**a**) 9 weeks of feeding (*n* = 8–9) and (**b**) 24 h after I/R injury (*n* = 6–7). For the latter, cardiomyocytes were isolated from the remote area. The speed of transient increase/decay reflects the fluorescence change (△F) during 75% increase/decay of the transient divided by the corresponding time period in s. Quantification of the contractile amplitude and the kinetics of sarcomere contraction/relaxation (**c**) prior (*n* = 8–9) and (**d**) post I/R injury (*n* = 8–9). Calcium cycling and sarcomere function of DD cardiomyocytes is enhanced at baseline, but deteriorated post I/R; *P ≤ 0.05, **P ≤ 0.01.

In a final set of experiments, we further aimed to address the biomechanical consequences of the large lipid load in cardiomyocytes of DD-fed mice before and after the insult. To this end, we analyzed measures of contractile function in control and prediabetic cardiomyocytes isolated from healthy (remote) myocardium of DD or chow mice after 9 weeks of feeding and 24 h after acute infarction. Interestingly, the massive lipid accumulation detected in prediabetic hearts after 9 weeks of DD feeding (Figs. 1 top and 2a) was associated with a small, but significantly enhanced calcium cycling in isolated cardiomyocytes (Fig. 8a). However, whereas cardiomyocytes from chow-fed animals exhibited an unchanged or only moderately increased calcium cycling 24 h after IR, prediabetic cardiomyocytes showed significant impairment in all parameters determined (Fig. 8b). Similar findings were obtained for the sarcomere function of cardiomyocytes. Prior induction of MI, both contraction and relaxation were increased in cardiomyocytes from DD mice, but post MI all measures collapsed, while control cardiomyocytes displayed a raise in both parameters as compared to baseline conditions (Fig. 8c, d).

Taken together, it seems that the high lipid load in prediabetic cardiomyocytes requires enhanced calcium cycling and sarcomere function to maintain normal cardiac performance, which both seem already at limit under baseline. This is additionally supported by challenging experiments with isoproterenol, in which prediabetic cardiomyocytes tended not to be able to increase their performance to the level of control cardiomyocytes (Supplementary Fig. 8). As a consequence, after MI, this also cannot be increased further resulting in a lower capability of the remote myocardium to compensate for the restricted contractility of the infarcted area.

## Discussion

In the present study, we used comprehensive in vivo MR techniques for non-invasive longitudinal deep phenotyping of multiple organs to monitor the transition from health to the prediabetic state, subsequent MI, and the following healing process. In the initial phase, we identified tibial intramyocellular lipid (IMCL) levels as most sensitive parameter to indicate very early alterations during diet-induced development of prediabetes, even before whole-body weight gain. During follow-up, we further revealed IMCLs together with subsequent cardiac lipid deposition as predictive markers for functional outcome after MI in prediabetic mice.

An association of increased IMCL levels and diabetes has already been described previously in both rodents and humans, but only in patients with overt type 1/2 diabetes^18,19^ or obesity^20^ and severely affected animal models, such as db/db^21^ or ob/ob^22^ mice and also Zucker diabetic fatty rats^23^. However, here we demonstrate that tibial IMCLs already rise at a very early stage of prediabetes. Of note, most likely this applies to all skeletal muscles, but only the tibial muscle allows a robust discrimination of EMCLs and IMCLs by localized spectroscopy^12^. Parallel multi-organ ^1^H MRS assessment revealed also succeeding increases in extramyocellular, hepatic, and cardiac lipids, while IMCLs remained at an unaltered high level over time (Figs. 1, 2). Thus, the initial IMCL increase may serve as an indicator of the subsequent lipid overload of other organs, which in case of a cardiovascular event, such as MI, renders the heart more vulnerable to adverse cardiac function. Even when the functional impact was small in our study and required regional analysis tools to be unveiled (Fig. 7), the consequences of the ectopic lipid accumulation may become even more severe in an advanced disease state of diabetes.

We found the diet-induced lipid load of the heart to be associated with substantial consequences on biomechanical properties of cardiomyocytes isolated from the healthy remote myocardium: Before induction of MI, prediabetic cardiomyocytes exhibited enhanced calcium cycling and sarcomere function at the limit, which could not be further increased after MI, resulting in a reduced ability of the remote myocardium to compensate for the impaired contractility of the infarcted area (Figs. 7, 8). The causative relationship between ectopic lipid deposition, altered calcium homeostasis, and sarcomere function is further supported by previous findings in db/db mice at an advanced diabetic state^24^: in that study, the massive lipid accumulation in db/db cardiomyocytes was found to be accompanied by a dysregulation of cardiomyocyte Ca^2+^ handling and an altered stiffness of myofilaments most likely mediated by a lipotoxic impairment of the SERCA^24–27^. Interestingly, similar alterations in Ca^2+^ sensitivity and cardiac myofilament function were also described in heart biopsies of T2D patients^28^. In this context, depressed SERCA activity and lower Ca^2+^ transients have as well been shown in other rodent diabetes models^29–31^. On the other hand, upregulated ceramides have been described to increase diastolic calcium in cardiomyocytes^32^—another putative cause for the reduced calcium cycling after MI. Accordingly, the increased cardiac ceramide levels (Supplementary Fig. 7) are likely to contribute to the larger functional impairment of DD hearts post I/R^33^. Of note, in the present study, prediabetic cardiomyocytes were characterized only one day prior/post induction of MI and might be even more impaired in the follow-up by the massive lipolysis which resulted in an almost complete depletion of the overloaded lipid pools in heart and liver until day 7 post-MI (Figs. 4, 5). Here, the released excess free fatty acids are well known to contribute to lipid-induced myocardial dysfunction after I/R^34–36^.

In contrast, the diabetes underlying low-grade inflammation seems to play only a minor role in the adverse outcome of diet-induced obesity (DIO) mice after MI—at least in the prediabetic state and the time points investigated in the present study. During the course of DD, we indeed found myeloid skewing of circulating and BM-derived immune cells (Fig. 3) supporting the previous assumptions of diabetes-mediated myelopoiesis^11,37^ and specifically leading here to moderate neutrophilia and monocytosis. However, ^19^F MR inflammation imaging provided no evidence for a severely altered immune response after MI (Fig. 6). Thus, it appears that DIO causes a mildly disturbed hematopoiesis under ‘plain’ feeding conditions, but this does not seem to affect immune cell release into the blood and subsequent recruitment into the infarcted myocardium after I/R.

Interestingly, the diet-induced prediabetic state with both impaired glucose tolerance and insulin sensitivity as well as massive ectopic lipid deposition did not affect cardiac function prior induction of MI. In fact, longitudinal in vivo monitoring showed that both global and regional cardiac function was still preserved after 9 weeks of DD-feeding compared to control diet (Figs. 6, 7, Supplementary Fig. 3). Nevertheless, we noticed at this time a kind of ‘overshoot’ in cardiomyocytic calcium handling and sarcomere function in DIO mice (Fig. 8), but which then collapsed after MI. Of note, in db/db hearts in the advanced diabetic stage, deteriorations of calcium transients, sarcoplasmic reticulum activity, and contractility have already been observed without any further cardiovascular intervention^38–40^. It seems, that in the prediabetic state cardiac function could yet be sustained, but after induction of MI the necessary compensatory mechanisms in the remote myocardium cannot be further exhausted. Importantly, the compensatory contractile function in the remote area after induction of MI was all the weaker, the higher the tibial IMCL content after 3 weeks of diet and the larger the subsequent increase in cardiac lipids after 9 weeks of diet (Fig. 7).

Alterations in IMCL content have been investigated in the context of a variety of diseases, but to the best of our knowledge neither an association of IMCL with the lipid burden of other organs nor their impact on the outcome after I/R has yet been systematically and longitudinally studied. The results of our study demonstrate that the initial IMCL increase indicates an enhanced vulnerability of the heart to subsequent lipid overload and dysfunction and, thus, may serve as an additional risk marker for an upcoming cardiovascular event. In contrast to common blood-based measurements as determination of free fatty acids, triglycerides, glucose tolerance, or insulin sensitivity, our approach provides direct access to affected organs and a metabolic profiling with regard to their successive lipid load, which then can be used for individual risk stratification. Nevertheless, further studies have to been carried out that the current observations in a well-controlled homogenous mouse cohort can be transferred to a patient population, with many other factors affecting organ lipids and cardiovascular health. However, beyond the heart, our findings may also apply for assessing the susceptibility of other organs, such as liver, kidney, etc. Finally, the non-invasive quantification of tissue lipids can help to identify prediabetic patients at an early stage allowing interventions already before the transition point to overt diabetes and to prevent the progression to T2D.

Taken together, persisting screening of organ lipids by ^1^H MRS—to also take into account interim changes upon dietary interventions or exercise^41,42^—may serve for risk stratification in patients, with tibial IMCLs, in particular, being easily measurable. Here, dedicated leg surface coils and the superficial anatomical position of the tibia will provide ultra-high sensitivity. Finally, the “leg-only” insertion into the front of the magnet ensures maximum patient comfort with minimal anxiety in the MR scanner.

## Methods

### Animal studies

All experiments were conducted in accordance with the guidelines for the use of laboratory animals under the German Animal Welfare Act and approved by the NRW State Office for Nature, Environment, and Consumer Protection (file reference 81-02.04.2018.A079 and 84-02.04.2015.A489). Male, 10 to 14-week-old C57BL/6J wild-type mice were purchased from Janvier Labs (Le Genest-Saint-Isle, France) and kept under standardized conditions in the central animal research facility of the Heinrich Heine University Düsseldorf at 22 ± 1 °C, with unlimited access to water and food with a 12 h light/12 h dark cycle.

### Diabetogenic diet

Starting at 10–14 weeks of age, age- and weight-matched male C57BL/6J mice were randomly assigned to one of two treatment groups receiving a high-calorie (high fat, high sugar) diet (DD, sniff Spezialdiät GmbH, DE-59494 Soest) or standard chow (control) for up to 11 weeks. Food consumption was measured in a subgroup of *n* = 5–7 mice over 8 days with values of 3.3 ± 0.4 and 3.0 ± 0.2 gram/day food intake for control and DD mice, respectively, with no significant differences between the groups. Blinded data collection and/or analysis was performed in the following experiments: ELISA, in vivo ^1^H MR measurements, flow cytometric analysis, calcium cycling, histological staining.

### Intraperitoneal glucose and insulin tolerance tests (i.p. GTT and i.p. ITT)

After starvation for 6 h, fasting blood glucose concentration was measured and mice were injected with 1 mg/g body weight of glucose (stock solution 40 g glucose monohydrate (w/v) in 100 ml water; sterile filtered) or 0.75 U/kg body weight of insulin. Blood glucose concentrations were measured basal and 5, 15, 30, 60, and 120 min after injection of the glucose or insulin bolus using an Accu-Check Compact Plus blood glucose meter (Roche Diagnostics, Mannheim, Germany).

### Plasma insulin analysis

To obtain blood plasma, the right ventricle of the heart was punctured and the blood obtained was anticoagulated with EDTA solution (100 mmol/l in isotonic sodium chloride solution). Plasma was collected after centrifugation at 3000 rpm, 4 °C for 15 min, followed by centrifugation at 13,000 rpm, 4 °C. Plasma insulin concentration was analysed using the ultra-sensitive insulin mouse ELISA Kit, Invitrogen (Thermo Fisher Scientific).

### Ischemia reperfusion injury (I/R)

Induction of cardiac I/R injury was essentially carried out as previously decribed^43^. In brief, mice were anesthetized with isoflurane (2–3%) and mechanically ventilated at a rate of 150 strokes/min and a tidal volume of 250–300 µl. Each animal was placed in a supine position with paws taped to an electrocardiogram (ECG) board (lead II) to confirm ST-segment elevation during MI. The chest was opened with a lateral cut along the left side of the sternum. Subsequently, the pericardium was gently dissected to allow visualization of coronary artery anatomy. Ligation was carried out for 50 min with an 8-0 polypropylene suture using a tapered needle passed underneath the left anterior descending artery (LAD), 1–3 mm from the tip of the left auricle. The success of infarction was verified microscopically by the absence of blood flow in the epicardium as well as significant elevation of the ST segment. The chest was then closed with 6-0 polypropylene suture with one layer through the muscle and a second layer through skin and subcutaneous tissue.

### Magnetic resonance imaging and spectroscopy (MRI + MRS)

All MR experiments were performed in vertical 9.4T Bruker AVANCE^III^ or NEO Wide Bore NMR spectrometers (Bruker) driven by ParaVision 5.1 and 360v3.6, respectively, and operating at a frequency of 400.21 MHz for ^1^H and 376.54 MHz for ^19^F. Data were acquired using Bruker Microimaging units (Micro 2.5) and actively shielded 40 mm gradient sets (1.5 T/m maximum gradient strength, 110 µs rise time at 100% gradient switching) essentially as described previously^44,45^. The animals were anesthetized with 1.5% isoflurane in air and to prevent drying of the mucous membranes, the anesthetic gas mixture was moistened. The front paws and the left hind paw were connected to ECG electrodes (CAS Medical Systems) and respiration was monitored using an air pillow on the animals’ back. Vital functions were recorded with an M1025 system (SA Instruments) and the body temperature was kept at 37 °C. Imaging/spectroscopy of heart and liver was carried out with 25 mm quadrature resonators optimized for cardiovascular applications^46^, while for the calf a 10 mm quadrature resonator was utilized (all Bruker).

#### Cardiac imaging

For analysis of heart function and infarct size, high-resolution images of mouse hearts were acquired by using an ECG- and respiratory-gated segmented fast gradient echo cine sequence with steady state precession (FISP): flip angle (FA) 15°, echo time (TE) 1.2 ms, 128 segments, repetition time (TR) 6–8 ms. Per heart cycle were 16 frames acquired with a field-of-view (FOV) of 30 × 30 mm^2^; matrix size (MS) 256 × 256; slice thickness (ST) 1 mm, acquisition time (TAcq) ∼1 min. Eight to ten contiguous slices were acquired to cover the entire heart. For evaluation of functional parameters (e.g., EDV, ESV, EF), ventricular demarcations in end-diastole and -systole were manually drawn with the ParaVision Region-of-Interest (ROI) tool (Bruker, Rheinstetten, Germany). For late gadolinium enhancement (LGE), a bolus of Gd-DTPA (gadolinium-diethylenetriaminepentacetate; 0.2 mmol Gd-DTPA per kg body weight) was applied intraperitoneally.

#### Tissue lipids by ^1^H MRS

For quantification of cardiac lipids, a 1 × 2 × 3 mm^3^ voxel was placed in the septum as shown in Fig. 1 top and Supplementary Fig. 2. Fieldmap-based shimming (MAPSHIM) was carried out to optimize the field homogeneity in the region of interest followed by manual shimming. ^1^H MR spectra were acquired using ECG- and respiratory-gated single-voxel point resolved spectroscopy (PRESS) with a chemical shift selective (CHESS) water suppression module and outer volume suppression (OVS). The following parameters were used: TR, 1000 ms; TE, 9.1 ms; averages, 1024; data points in the spectral domain, 256; spectral width, 5000 Hz; acquisition time, 51.2 ms; an exponential filter of 10 Hz was applied and chemical shifts were referenced to the prominent triglyceride methylene (-CH_2_-) peak in the water-suppressed spectra at 1.3 ppm. The exact repetition time of the sequence was determined by the heart and respiration rate of individual mice. Total preparation time including CHESS (73.23 ms) and OVS (18.63 ms) was 91.86 ms. Localized acquisition was timed at ∼40% of the cardiac cycle in systole to maximize tissue thickness and homogeneity, typically requiring a trigger delay of 60–80 ms after ECG R-wave upslope detection (see also Supplementary Fig. 2). To quantify the myocardial metabolite content, the integral of the lipid signal was normalized to the total creatine content. Of note, the calculated data may be influenced by unknown partial saturation factors due to an unknown effective TR, which can introduce a bias in case the heart rate consistently differs between groups. However, Supplementary Fig. 9 demonstrates that heart rates of anaesthesized mice during the ^1^H MRS measurements were unaltered between the DD and control groups over time both during 9 weeks of feeding as well as 3 weeks follow-up upon I/R.

For analysis of liver fat, localized respiratory-triggered ^1^H MR spectra were acquired from a 2 × 2 × 2 mm^3^ voxel placed in the upper part of the left liver lobe (Fig. 1, middle) using essentially the same shimming procedures and acquisition parameters as described above. Spectra were recorded with a PRESS sequence without water suppression but with OVS (256 averages, 2048 data points, TAcq, 4 min). Exponential weighting resulting in a 20 Hz line broadening was applied before Fourier transformation. Chemical shifts were referenced to the water signal at 4.7 ppm. After manual phase and baseline correction, signals arising from water and lipid protons were integrated for quantification of the liver fat content. The results are given as a percentage of the total ^1^H signal.

For determination of *tibial intra- and extramyocellular lipids* (IMCL and EMCL), mice were positioned on their left side within the animal handling system and the right calf was fixed in the 10 mm resonator with the tibialis anterior (TA) muscle aligned along the main magnetic field direction ensuring maximal spectral separation of IMCL and EMCL resonances^12^. The spectroscopic voxel (1.3 × 1.3 × 3 mm^3^) was carefully placed within the TA muscle to avoid any contaminations by subcutaneous fat (Fig. 1, bottom). Shimming was carried out as described above and localized ^1^H MR spectra were acquired using a PRESS sequence with OVS (TR, 1000 ms; TE, 20 ms; spectral bandwidth, 5000 Hz; data points, 2048; TAcq, 18 min; averages, 1024). For water suppression variable power radiofrequency pulses with optimized relaxation delays (VAPOR) were used. An exponential filter of 2 Hz was applied and the methyl signal of creatine (Cr) at 3.02 ppm was used as concentration reference, i.e., IMCL and EMCL levels in TA muscle are given as intensity ratios IMCL/Cr and EMCL/Cr, respectively.

Water line widths were around 15–30 Hz for the myocardium, 30–50 Hz for the liver, and 10–20 Hz for the tibial muscle. Spectral analysis was carried out with TopSpin (Bruker) which was used for Fourier transformation, phase and baseline correction, deconvolution and integration. Quantifications were not corrected for partial saturation effects.

#### T2 mapping and water/fat separation

For the calf, additionally water/fat separation by multi chemical shift selective imaging^13,47^ (mCSSI) and T2 mapping^48^ was used for further tissue characterization employing the same FOV and matrix size (25.6 × 96 mm^2^; 256 × 96) for both approaches. For T2 mapping, 16 echoes were acquired with a spacing of 5.13 ms (TR 500 ms, averages 6, TAcq 4 min 48 s); for water/fat separation, chemical shift selective images were taken at 4.7 and 1.3 ppm, respectively (TR 2000 ms, TE 4.8 ms, RARE factor 8, averages 4, TAcq 1 min 36 s).

#### ^1^H/^19^F MR inflammation imaging

For ^19^F labeling of circulating immune cells^16,49^, mice were anesthetized (1.5% isoflurane) one day after induction of MI and 3 mmol per kg body weight perfluorocarbons (PFCs) were injected i.v. into the tail vein. Forty-eight hrs later, ^1^H/^19^F MRI experiments were performed using a 25 mm quadrature ^19^F resonator (Bruker) optimized for cardiovascular applications (see above) with one channel tunable to both ^1^H and ^19^F. ^19^F RARE: TR = 2500 ms, TE = 3.09 ms, RARE factor 32, FOV 2.56 × 2.56 cm^2^, matrix 128 × 128, ST 1 mm, TAcq 20 min. For a detailed description of the entire ^19^F approach (validation, data analysis etc.) please refer to refs. ^16,50^.

### Flow cytometry

Single cell suspensions of blood and stromal vascular fraction (SVF) of bone marrow (BM) were obtained from mice fed either with DD or chow for 9 weeks. All samples were analyzed on a BD Biosciences (San Jose, CA, USA) LSR-Fortessa^TM^ flow cytometer. Absolute cell counts were determined using Flow-Count Fluorospheres (Beckman Colter Inc, Krefeld, Germany). Kaluza flow analysis software (Beckman Colter Inc) or FlowJo software (Treestar, San Carlos, CA) was used for subsequent data analysis. All antibodies and corresponding clones are listed in Supplementary Table 1; gating schemes for analysis of cells obtained from blood and bone marrow are provided in Supplementary Figs. 10, 11, respectively.

#### Blood

Single cell suspensions were obtained from tail vein blood using EDTA Microvettes® (Sarstedt, Germany). To avoid unspecific antibody binding to Fc receptor expressing cells, all samples were incubated with purified recombinant Fc protein (Fc block, BioLegend, San Diego, USA) for 10 min at 4 °C. Single cell suspensions were then stained with different antibodies (15 min, 4 °C, light protected) using fluorophore-coupled markers. Hypotonic lysis buffer (155 mM NH_4_Cl, 10 mM KHCO_3_, 0.1 mM EDTA; Sigma-Aldrich Invitrogen, Carlsbad, CA, USA) was then added for 7 min at 4 °C to lyse the erythrocytes and exclude non-specific antibody binding to the erythroid cells. Cell sedimentation was performed at 800 × *g* for 10 min at 4 °C, the cell pellet was resuspended in 50 µl PEB buffer and fixed with 1% (v/v) Roti®-Histofix (Carl Roth GmbH & Co KG, Karlsruhe, Germany) for 20 min at RT. The fixative was removed by final centrifugation (800 × *g*, 4 °C, 10 min), followed by addition of PEB buffer.

#### Bone marrow SVF

BM SVF was enzymatically digested with 1 ml digestion solution for 20–30 min at 37 °C. Single cell suspension was then filtered through a 70 µm nylon filter (Greiner Bio-One, Frankfurt, Germany), centrifuged at 300 × *g* for 10 min at 4 °C and collected in PEB. To avoid unspecific antibody binding to Fc receptor expressing cells, all samples were incubated with purified recombinant Fc protein (Fc block, BioLegend, San Diego, USA) for 10 min at 4 °C. LIVE/DEADTM fixative was then added for 30 min at 4 °C to label dead cells. Next, single cell suspensions were stained with different antibody combinations (15 min, 4 °C, light protected) using fluorophore-coupled markers. After centrifugation, the samples were collected in PEB and fixed with 1% (v/v) Roti®-Histofix (Carl Roth GmbH & Co KG, Karlsruhe, Germany) for 20 min at RT. The fixative was removed by final centrifugation (800 × *g*, 4 °C, 10 min), followed by addition of PEB buffer.

### Hematoxylin/eosin (H/E) staining and determination of adipocyte size

For histochemical analysis, tibiae were removed from mice and fixed in 10% neutral buffered formalin for 48 h, decalcified in 25% EDTA buffer for 2 weeks, dehydrated and embedded in paraffin. A LEICA RM2255 rotary microtome (Leica Biosystems GmbH, Nussloch, Germany) was used to prepare 5 µm thick serial sections of the tibia. Two sections each were imaged on SuperFrost®Plus slides. In order to obtain a representative image and analysis of the bone marrow, the paraffin sections were not mounted until the entire bone marrow (BM) of the lower leg had been sectioned. After one day, the tissue was heat fixed at 60°C for one hour and then stored at RT for histochemical staining. Sections were deparaffinized through a graded ethanol series. First, the slides were incubated three times for 15 min with Roticlear® (Carl Roth GmbH & Co. KG, Karlsruhe, Germany) and then mixed with 100%, 96% and 70% ethanol for 2 min each time. The tibia longitudinal sections were then washed three times for 5 min with PBS. The sections were stained with H&E. BM adipocytes were quantified based on a previously calculated area (mm^2^) in the tibia. All microscopic images were acquired using a Zeiss AxioObserver.Z1 microscope and processed using ZEN 2 software (Carl Zeiss Microscopy GmbH). ImageJ software (NIH) was used to quantify the number and size of adipocytes in the BM.

### Liquid chromatography of ceramides with tandem mass spectrometry (LC‐MS/MS)

Cardiac tissue samples were extracted by precipitation in methanol. Internal standard (1 µM C17 S1P in MeOH, 10 µl) was added to weighted heart tissue, homogenized in MeOH (1 ml), and precipitated at −80 °C overnight. Tissue sample supernatants were concentrated in a vacuum rotator (60 °C, 1 h), the residue dissolved in MeOH (100 µL) and transferred into mass spectrometry vials. Chromatographic separation was performed on an LCMS-8050 triple-quadrupole mass spectrometer (Shimadzu Duisburg, Germany) interfaced with a dual ion source and a Nexera X3 front-end-system (Shimadzu Duisburg, Germany). High-performance liquid chromatography was performed with a 60 × 2 mm MultoHigh-C18 RP column with 3 µm particle size at 40 °C (CS Chromatographie Service, Langerwehe, Germany). Mobile phases consisted of [A] = MeOH and [B] = 1% (v/v) aq. HCOOH. The following LC gradient settings were used: nebulizer: 3 L/min, interface temperature 300 °C, desolvation temperature 526 °C, heat block temperature 400 °C and drying gas flow 10 L/min. The flow rate was 400 µL/min, and injection volume 10 µL (supplemental Table 1). Standard curves were generated by measuring increasing amounts (10 fmol to 50 pmol) of S1P and internal standard (C17 S1P, 0.1 µM final conc. in MeOH). The linearity of the standard curves and correlation coefficients were obtained by linear regression analysis. Data were collected using multiple reaction monitoring (MRM). Data analysis was performed with LabSolutions 5.114 (Shimadzu, Kyoto, Japan).

### Cardiomyocyte isolation

Hearts were quickly excised and washed in PBS before digestion by retrograde perfusion with collagenase type I (Worthington Biochemical Corp., Lakewood, NJ, USA) for 6 min at 37 °C as described previously^24^. Next, atria, right ventricles and all myocardium supplied by the LAD were trimmed off and only the remaining areas of the LV were used for further myocyte isolation. Tissue was minced and gently triturated using a Pasteur pipette to break down remaining tissue bits before pouring through a nylon mesh (150 μm pores) and stepwise recalcification of the cell suspension.

### Measurements of Ca^2+^ cycling and sarcomere length in single myocytes

Single myocytes isolated from mouse left ventricles were loaded with the fluorescent Ca^2+^ indicator Fura-2 for 15 min, washed 3 times and superfused with prewarmed buffer (in mM: 137 NaCl, 5.4 KCl, 1.2 CaCl_2_, 1 MgCl_2_, 10 HEPES, 5.5 glucose; pH 7.4; 37 °C). A dual excitation fluorescence imaging/contractility recording system (HyperSwitch Myocyte System, IonOptix LLC, Westwood, MA, USA) was used for measurements of Ca^2+^ transients in electrically paced cells (0.5 Hz) at excitation wavelengths 340 and 380 nm. Ratiometric data (340 nm/380 nm) obtained at a temporal resolution of 250 pairs/s were normalized for background fluorescence and analyzed using IonWizard software (Version 6.4, IonOptix). For each cell, analyses of 10 independent Ca^2+^ transients were averaged and the data of approximately 10 myocytes determined the value for one heart. The kinetics of Ca^2+^ changes were assessed as the fluorescence change over time. The speed of Ca^2+^ transient increase was calculated from the change of fluorescence (ΔF(340 nm/380 nm)) between the start of Ca^2+^ increase and 75% of Ca^2+^ transient peak divided by the duration of this time period (s). Analogously, the speed of Ca^2+^ transient decay was calculated from the change of fluorescence (ΔF(340 nm/380 nm)) between the Ca^2+^ transient peak and 75% of Ca^2+^ transient decay divided by its duration (s). For β-adrenergic stimulation, myocytes were superfused with buffer containing 10^−7^ M isoproterenol. Since buffers need to be prepared fresh every day, data from one mouse of each experimental group were recorded the same day. For measurements of sarcomere mechanical properties, sarcomere length was measured continuously in paced cardiomyocytes using a video detection system that recognizes the alternating dark and light A- and I-bands of the sarcomeres in a defined region of interest within a myocyte (MyoCam-STM, IonOptix). The density traces were converted to average sarcomere lengths at sampling rates of 240 Hz. The analysis of the contractile amplitude and the velocity of sarcomere contraction and relaxation was performed using IonWizard software (Version 6.4, IonOptix) analogous to the above described analysis of Ca^2+^ transients.

### Statistical analysis

Data were analyzed using Graphpad Prism 9 software (La Jolla, CA, USA) or OriginPro 2021 (Originlab Corporation, Wellesley Hills, USA). All data listed are presented as mean ± SD. Grubb’s test (*α* = 0.05) was used to determine outliers and exclude them from the cohort. According to this procedure the following outliers were detected: calcium measurement (one mouse) and insulin measurement (one mouse). Furthermore, due to technical issues one mouse was excluded from the quantification of the adipocyte size. Data were analyzed for normal distribution and for comparison of two experimental groups the unpaired Student’s *t* test was used. Two-way ANOVA was used to evaluate diabetogenic phenotyping over the time of feeding, myocardial infarction and the subsequent healing period. Significance was assumed at a *P* ≤ 0.05.

## Supplementary information

Supplementary material

## Data Availability

All data supporting the findings of this study are available within the article and its supplementary material. Raw MRI/MRS data are available from the corresponding authors.
