# Publisher Correction: Magnetic resonance reveals early lipid deposition in murine prediabetes as predictive marker for cardiovascular injury

**DOI:** 10.1038/s44303-024-00050-2

**Published:** 2024-10-11

**Authors:** Katja Heller, Vera Flocke, Tamara Straub, Zhaoping Ding, Tanu Srivastava, Melissa Nowak, Florian Funk, Bodo Levkau, Joachim Schmitt, Maria Grandoch, Ulrich Flögel

**Affiliations:** 1 Institute for Translational Pharmacology, Medical Faculty, University Clinics and Heinrich Heine University, Düsseldorf, Germany; 2 Experimental Cardiovascular Imaging, Institute for Molecular Cardiology, Heinrich Heine University, Düsseldorf, Germany; 3 Institute for Pharmacology, Heinrich Heine University, Düsseldorf, Germany; 4 Institute for Molecular Medicine III, Heinrich Heine University, Düsseldorf, Germany; 5 Cardiovascular Research Institute Düsseldorf (CARID), Heinrich Heine University, Düsseldorf, Germany

**Keywords:** Imaging techniques, Molecular imaging, Magnetic resonance imaging, NMR spectroscopy

Correction to: *npj Imaging* 10.1038/s44303-024-00044-0, published online 23 September 2024

“Acknowledgements were updated to include Simone Gorressen and to include funding from Deutsche Forschungsgemeinschaft (DFG, German Research Foundation). The original article has been corrected.”

