# Supplementary material for: Magnetic resonance reveals early lipid deposition in murine prediabetes as predictive marker for cardiovascular injury
Source: Npj Imaging. 2024 Sep 23;2:36. doi: 10.1038/s44303-024-00044-0 (PMC12118699; doi:10.1038/s44303-024-00044-0)
Supplement: Supplementary file 1 — Supplementary material [file 44303_2024_44_MOESM1_ESM.pdf]

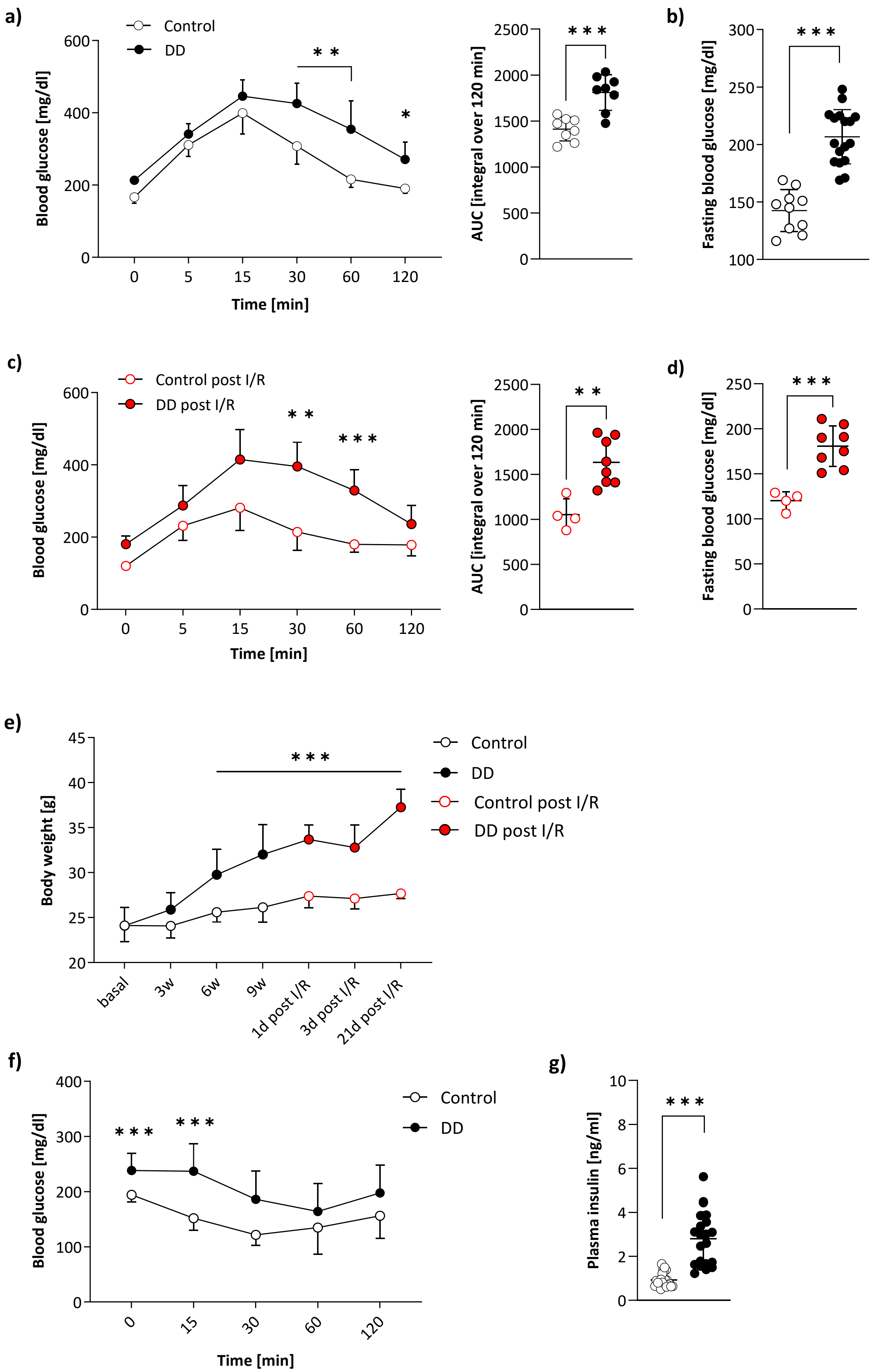

Supplementary Figure 1: Development of a prediabetic phenotype over time

a-d, Blood glucose levels of mice fed a control diet (open symbols) or DD (closed symbols) upon intraperitoneal glucose tolerance test (**a+c**, showing time course and AUC, i.e. the integral over 120 min) or fasting (**b+d**) after 9 weeks of feeding (**a+b**, n = 7-8) and additional 3 weeks post I/R (**c+d**, n = 4-8). **e,** Body weight of both groups over the entire observation period (n = 8 each). **f**, Blood glucose levels after intraperitoneal insulin tolerance test over time (n = 8 each) and **g**, plasma insulin values (n = 21-22) of mice fed DD versus control diet for 9 weeks; ***P*≤0.01, ****P*≤0.001.

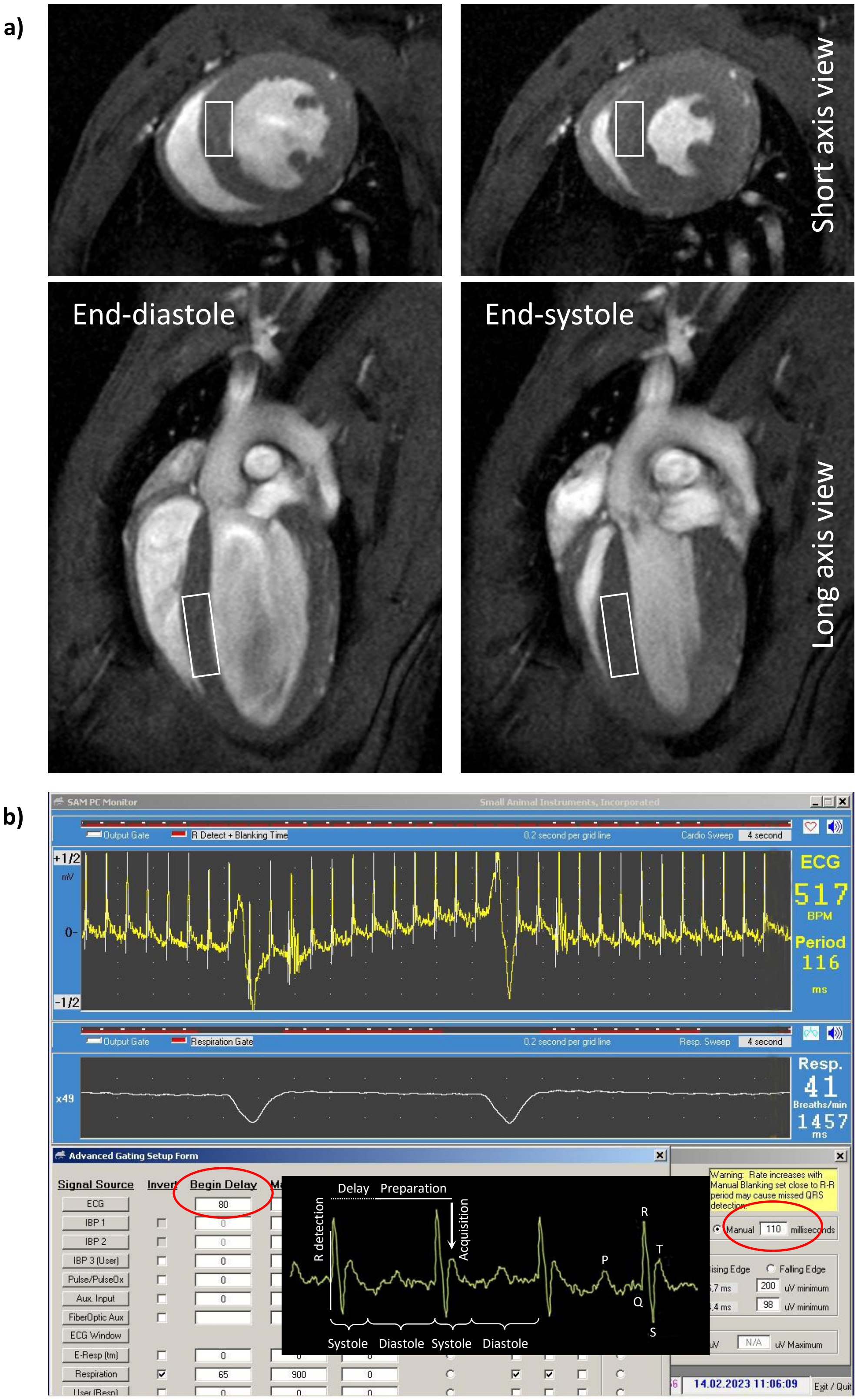

Supplementary Figure 2: Localization and timing for cardiac ^{1}H MRS

a Using end-diastolic and end-systolic short and long axis images, the spectroscopic voxel (1×2×3 mm^3) was carefully placed in the septum. This is where the least movement of the myocardium occurs over the entire cardiac cycle. Of note, during systole the voxel is completely surrounded by myocardium, ensuring the greatest possible homogeneity and minimizing contaminations from chamber blood or epicardial fat. In addition, coronary blood flow almost completely ceases during systole, which further helps to improve homogeneity in the region of interest. Thus, data acquisition in end-systole at the time of maximum septum thickness and homogeneity will be beneficial in terms of both spectral quality and myocardial specificity. **b** Since the total preparation time for water and outer volume suppression comprises 91 ms, the trigger signal to the spectrometer was delayed by approximately 60-80 ms (depending on the current heart rate) after detection of the R-wave in the ECG in order to perform data acquisition mainly during end-systole of the next heart cycle. Furthermore, the blanking time was set to approximately 95% of the cardiac cycle to avoid false-positive registration of a cardiac trigger signal.

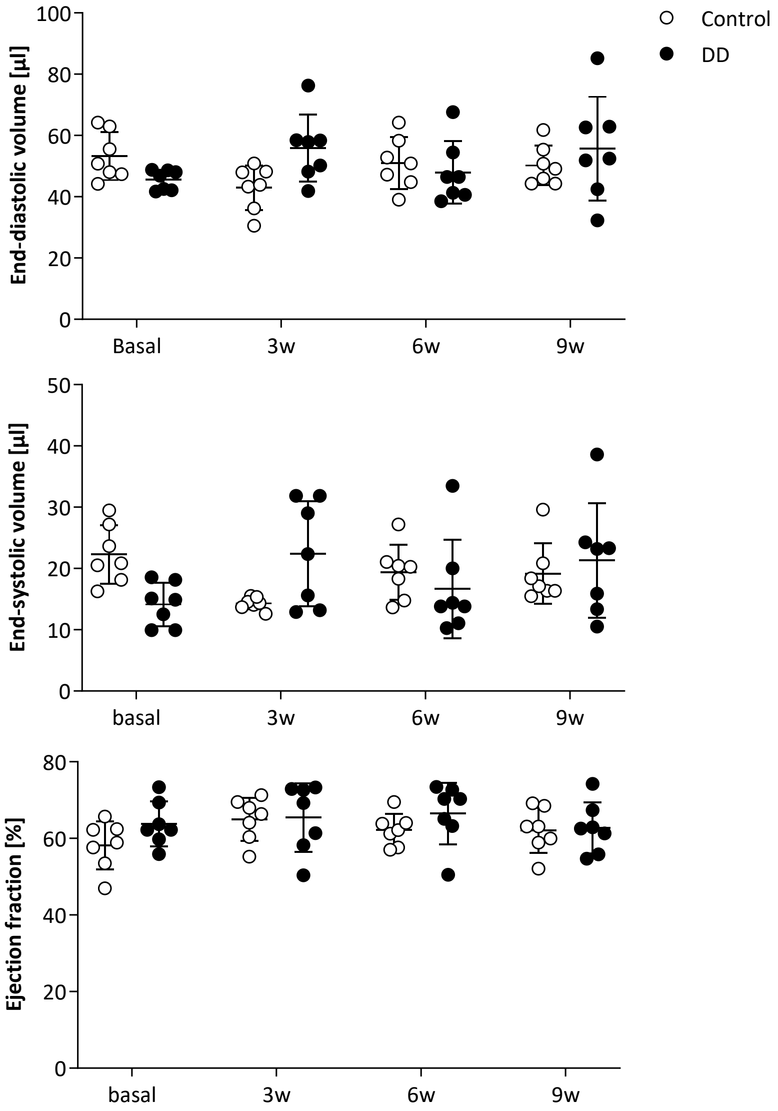

Supplementary Figure 3: Minor impact of DD on global cardiac function

Quantification of global EDV (**top**), ESV (**middle**), and EF (**bottom**) from cine ^{1}H MR movies after 9 weeks of feeding with open symbols for control diet and closed symbols for DD (n = 7 each).

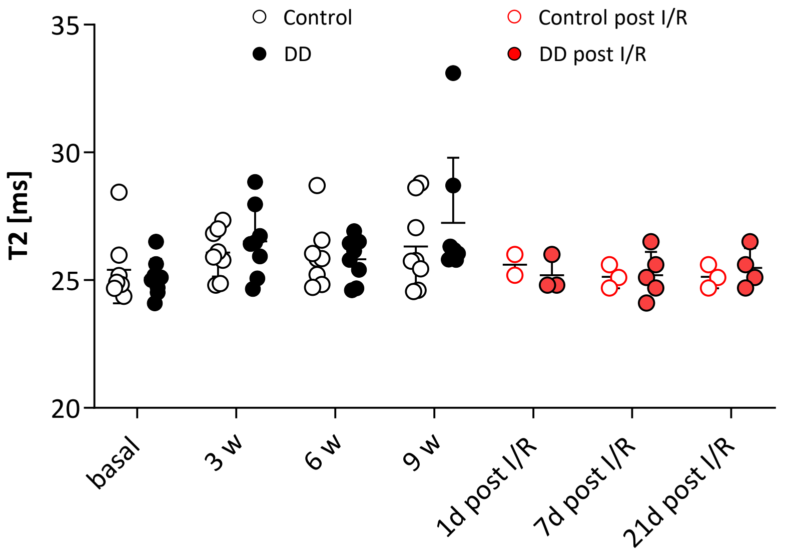

Supplementary Figure 4: Almost unchanged T2 of tibialis anterior muscle over the entire observation period

Quantification of T2 of tibialis anterior muscle during 9 weeks of feeding (black) and 3 weeks follow-up upon I/R (red) with open symbols for control diet and closed symbols for DD; n = 3-8.

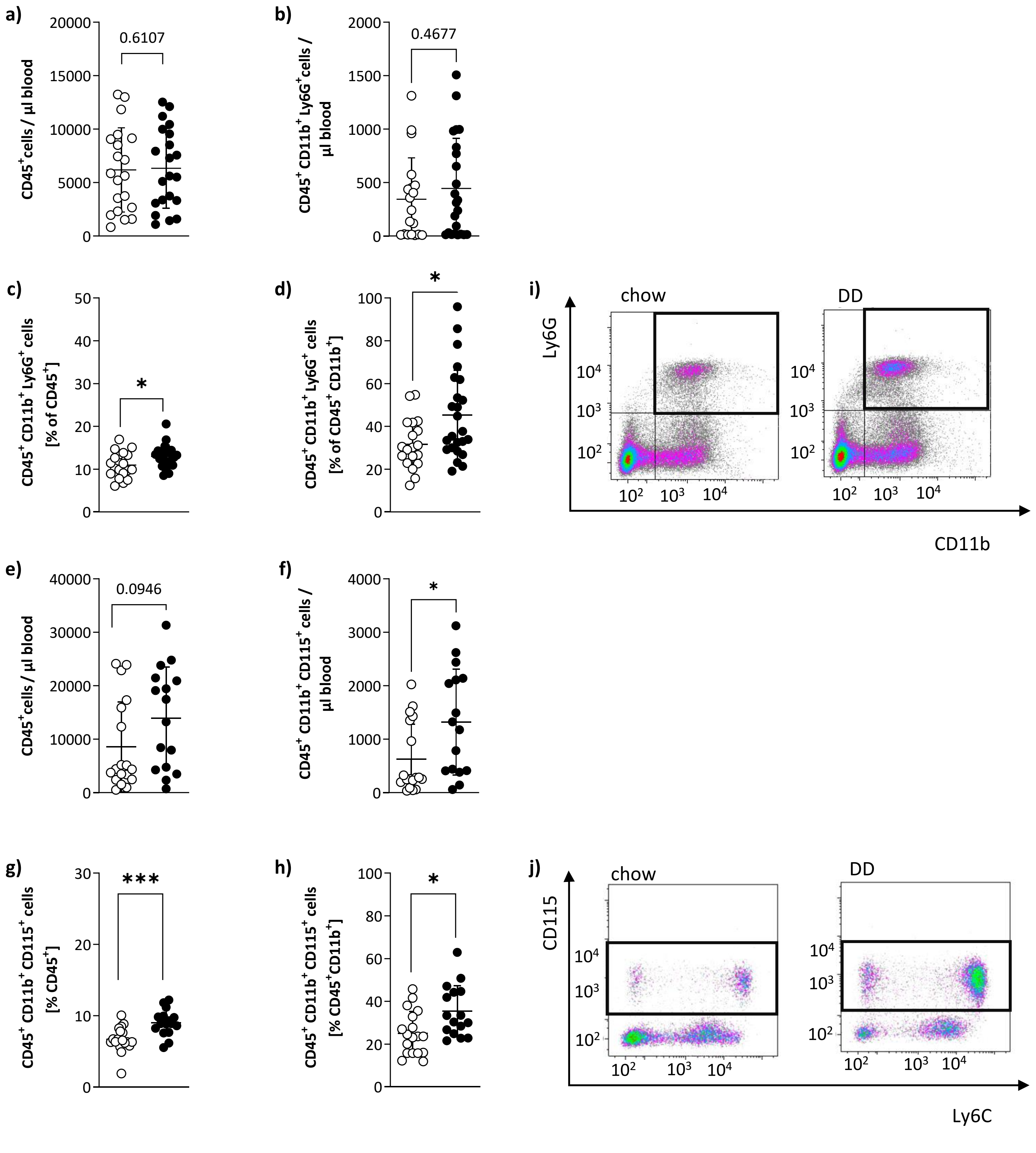

Supplementary Figure 5: Neutrophilia and monocytosis as an effect of DD feeding

Flow cytometric analysis of blood neutrophils (**a-d, i**) and monocytes (**e-h, j**) from mice fed DD and control diet. **a**, $CD45^+$ leukocytes per µl blood and **b**, absolute number of neutrophils per µl blood. **c**, Quantification of blood $CD45^+$ $CD11b^+$ $Ly6G^+$ neutrophils normalized to $CD45^+$ leukocytes and **d**, $CD45^+$ $CD11b^+$ cells (n = 21-23). **e**, $CD45^+$ leukocytes per µl blood and **f**, absolute number of monocytes per µl blood. **g**, Quantification of blood $CD45^+$ $CD11b^+$ $CD115^+$ monocytes normalized to $CD45^+$ leukocytes and **h**, $CD45^+$ $CD11b^+$ cells (n = 16-18). **i**+j, Representative density plots of the flow cytometric measurement of neutrophils and monocytes after DD and control, respectively; *$P \leq 0.05$, ***$P \leq 0.001$.

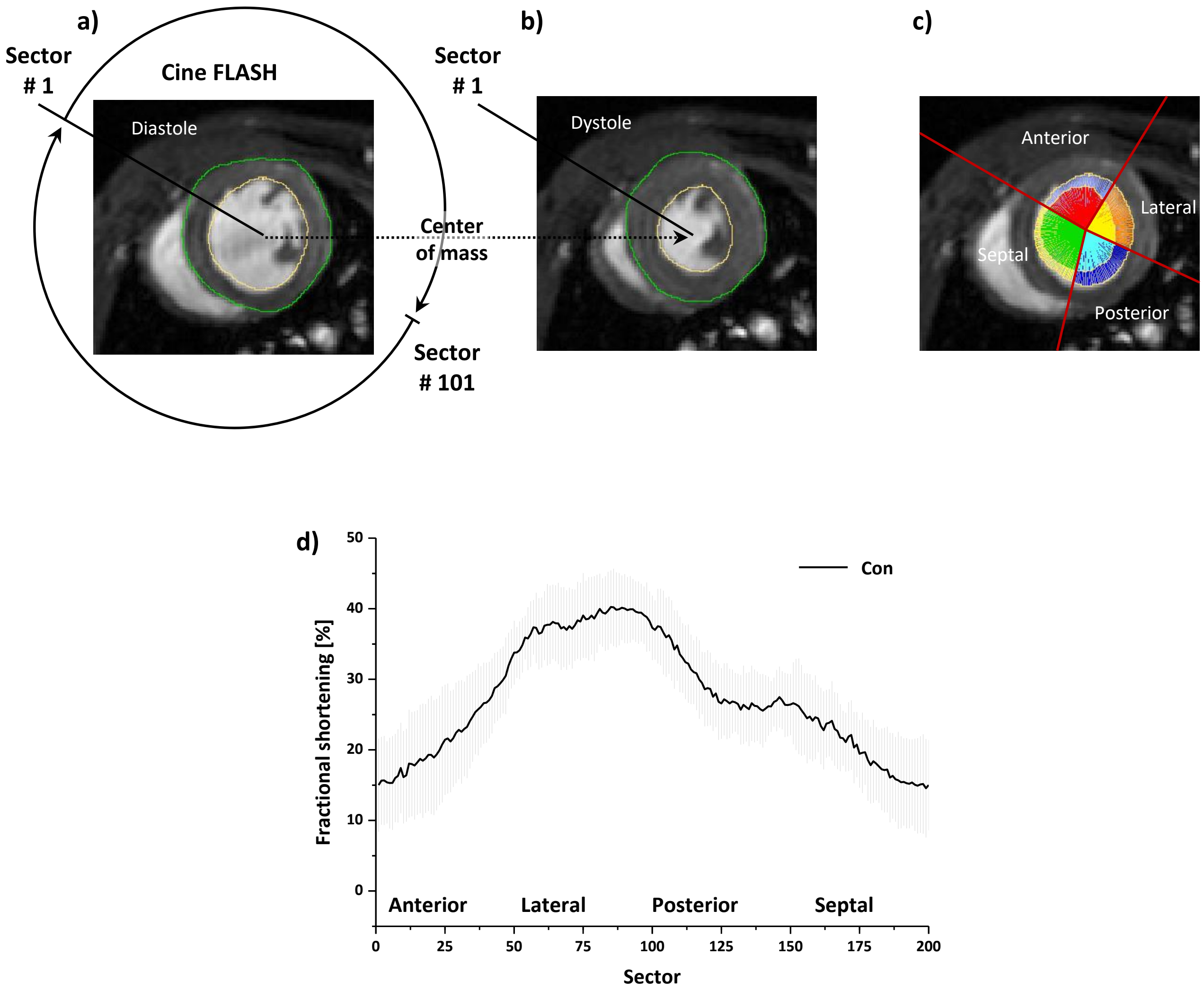

Supplementary Figure 6: Regional wall movement analysis over 200 sectors

Local fractional shortening (FS) was calculated from cine loops acquired in short-axis slices orientation. End-diastolic (**a**) and end-systolic (**b**) borders of the cavity were delineated and inner radii starting from the end-diastolic centre of mass were calculated before and after contraction. The left ventricle was then divided into 200 equally distributed sectors starting from the anterior insertion of the right ventricle (sector #1). Thereafter, FS was automatically calculated in each sector by an in-house developed software as the difference of end-diastolic and end-systolic radii divided by the end-diastolic value and displayed in radial charts (**c**). Note, that sectoral wall contraction can easily be assessed visually from the distance between diastolic and systolic endocardial radii in the radial FS chart. As expected, FS strongly depends on wall region. (**d**) Averaging over the indicated sectors demonstrated largely different values for FS over the different wall areas, with decreasing FS from lateral, posterior, anterior to septal sectors. Data are means $\pm$ SD from n=9 control animals. For sake of clarity, SDs are shown within the full sector plots as shadows. Adapted from references[43,48].

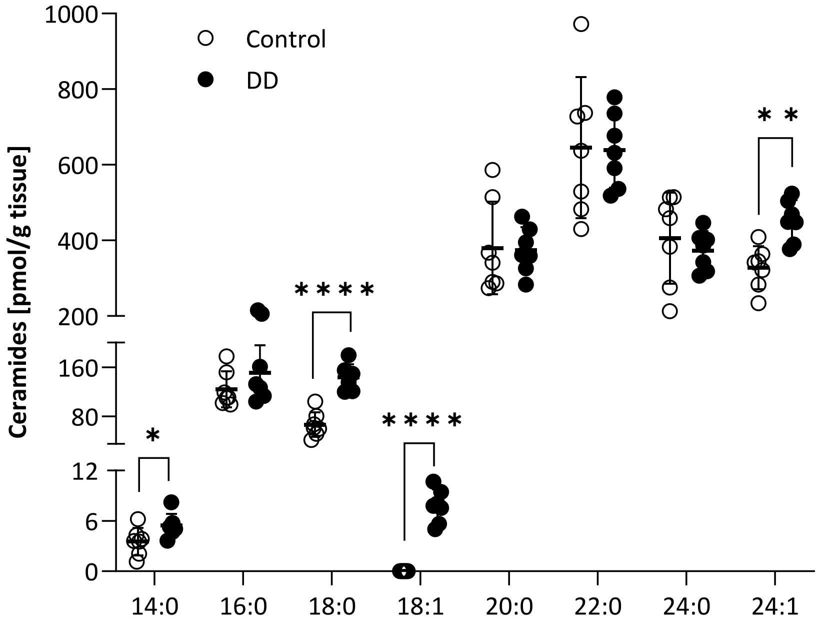

Supplementary Figure 7: Accumulation of several ceramide species in cardiac tissue of DD mice

Ceramide lipidomic profile of hearts from DD versus normal control mice after 12 weeks of diet. Data are presented as mean ± SD. Statistical analysis for each ceramide species was performed using unpaired two-tailed Student's t-test; n = 7 each; *P≤0.05, **P≤0.005, ***P≤0.001.

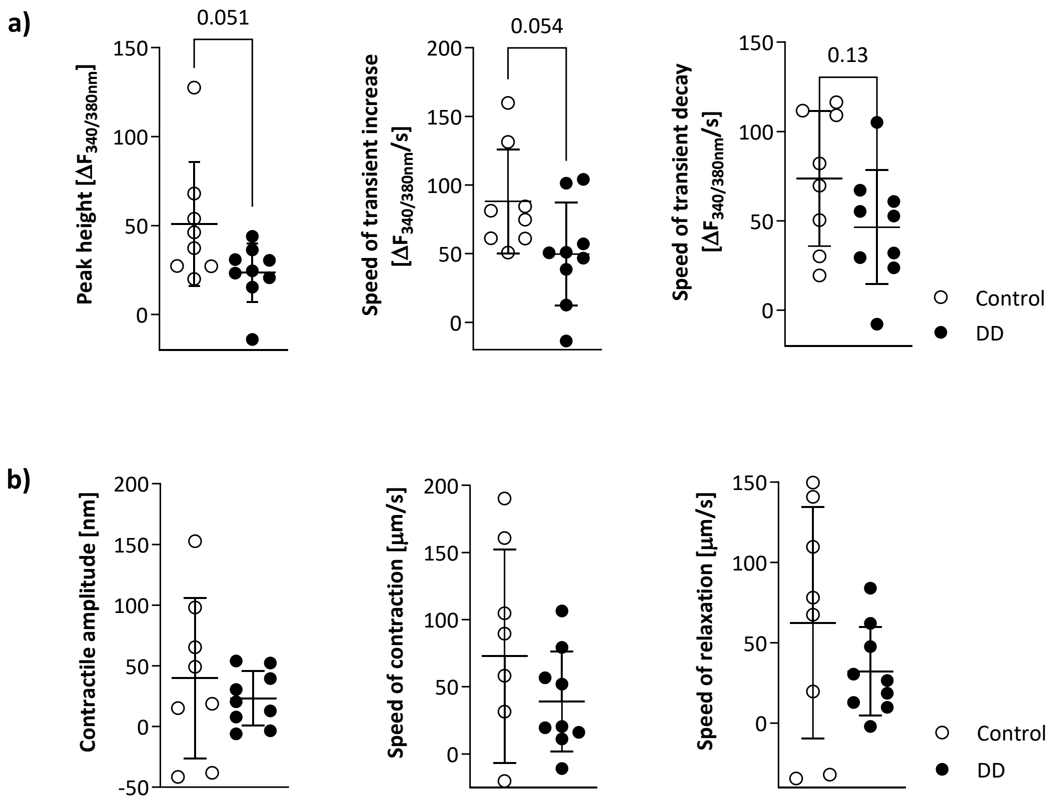

Supplementary Figure 8: Impaired enhancement of calcium cycling and sarcomere function in DD mice upon isoproterenol challenge

a, Percentage alterations in calcium cycling of isolated cardiomyocytes of DD (closed symbols) and control (open symbols) mice after 9 weeks of feeding upon exposure to isoproterenol (n = 8-9). **b**, Percentage alterations in the contractile amplitude and the kinetics of sarcomere contraction/relaxation upon exposure to isoproterenol (n = 8-9). The rise of calcium cycling and sarcomere function in DD cardiomyocytes is limited, yet without reaching the level of statistical significance.

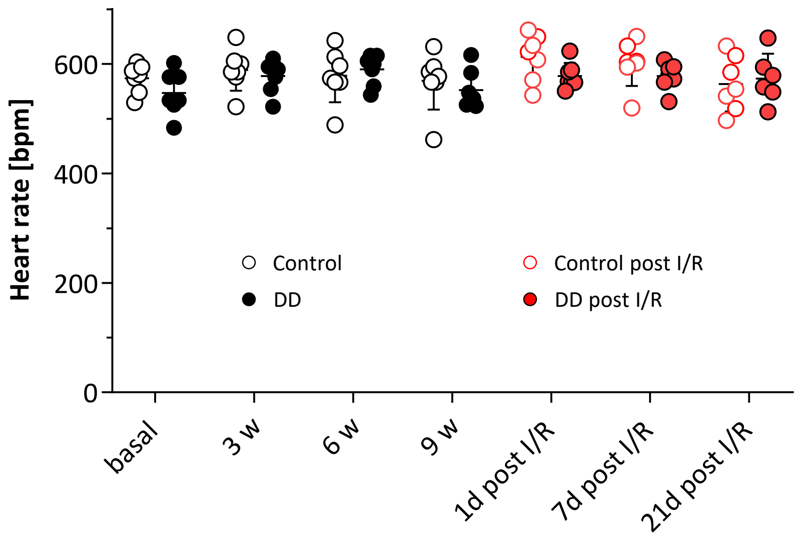

Supplementary Figure 9: Unchanged heart rates during ^{1}H MRS over the entire observation period

Heart rates of mice under anaesthesia within the magnet during 9 weeks of feeding (black) and 3 weeks follow-up upon I/R (red) with open symbols for control diet and closed symbols for DD; n = 6-7.

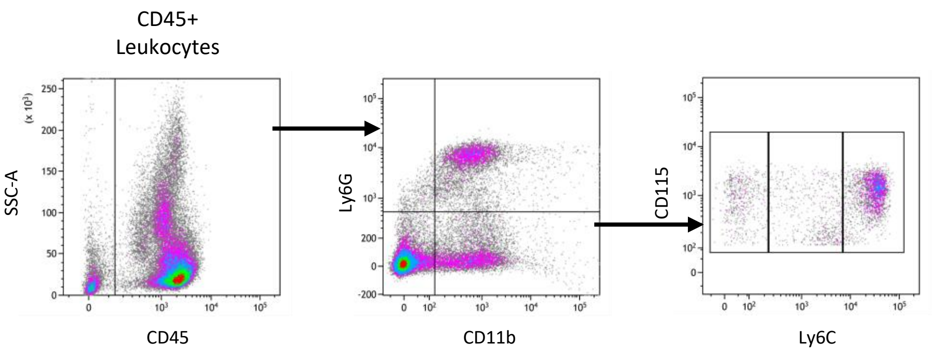

Supplementary Figure 10: Gating scheme for the analysis of neutrophils and monocytes in murine blood

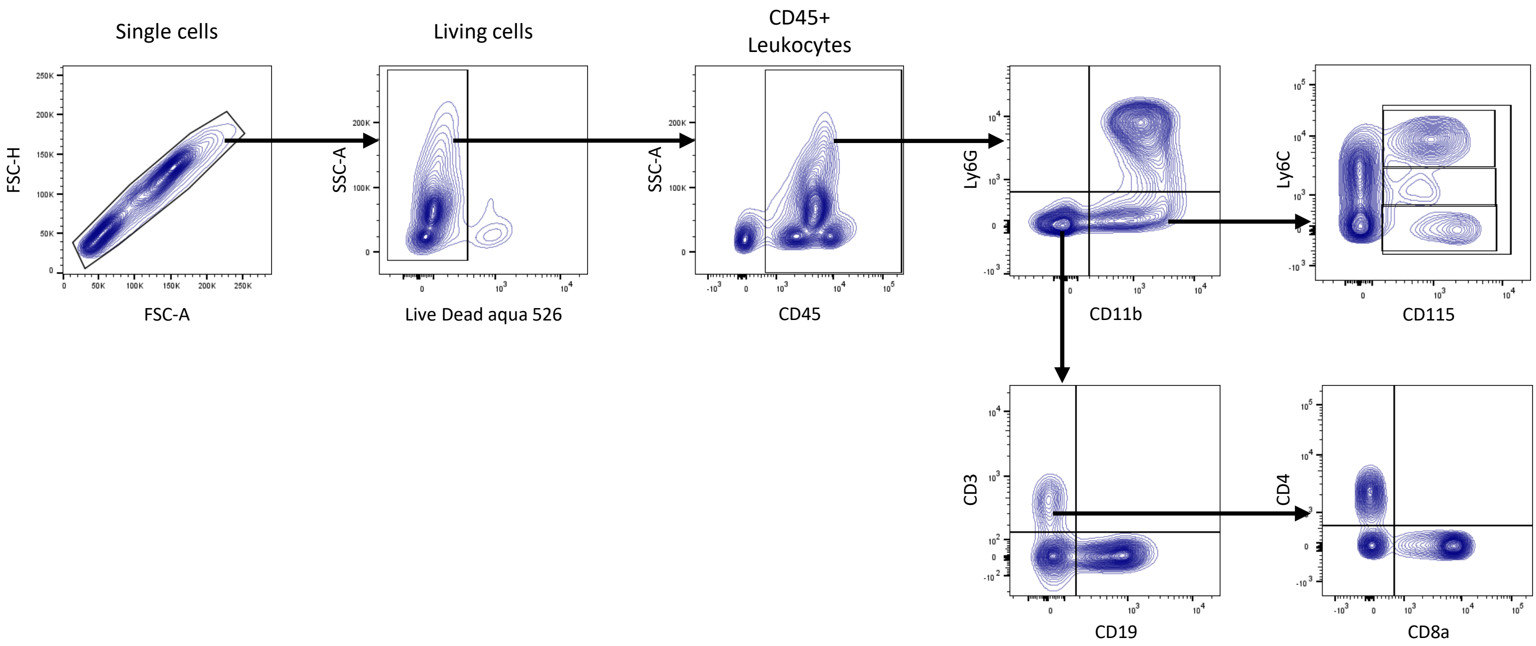

Supplementary Figure 11: Gating scheme for the analysis of bone marrow-derived cells

Blood monocytes and neutrophils

Antibody	Clone	Cat. No.	Vendor	Concentration
CD45-AF700	30-F11	103106	Biolegend	1:25
CD11b-PE	M1/70	101207	BD Bioscience	1:25
CD11c PE-Cy7	N418	117317	Biolegend	1:25
Ly-6G-PacBl	1A8	127611	Biolegend	1:25
CD115-APC	AFS98	17115282	Invitrogen	1:25
Ly-6C-AF488	HK1.4	128021	Biolegend	1:25

Bone marrow mature leukocyte markers

Antibody	Clone	Cat. No.	Vendor	Concentration
CD19-PacBl	6D5	115523	Biolegend	1:12.5
Ly6G-BV650	1A8	127641	Biolegend	1:12.5
CD45-PE	30-F11	103106	Biolegend	1:25
CD3-AF700	17A2	100216	Biolegend	1:50
CD8a-AF647	53-6.7	100724	Biolegend	1:50
CD115-BV711	AFS98	135515	Biolegend	1:50
F4/80-BV605	BM8	123133	Biolegend	1:50
CD11b-PE/Dazzle	M1/70	101256	Biolegend	1:100
CD4-FITC	RM4-5	MCD0401	LifeTechnologies	1:200
Ly6C-APC/Cy7	HK1.4	128026	Biolegend	1:200

Supplementary Table 1: Antibodies for flow cytometry
